# PIABC: Point Spread Function Interpolative Aberration Correction

**DOI:** 10.3390/s25123773

**Published:** 2025-06-17

**Authors:** Chanhyeong Cho, Chanyoung Kim, Sanghoon Sull

**Affiliations:** School of Electrical Engineering, Korea University, 145 Anam-ro, Seongbuk-Gu, Seoul 02841, Republic of Korea; chcho@mpeg.korea.ac.kr (C.C.); cykim@mpeg.korea.ac.kr (C.K.)

**Keywords:** CMOS image sensor, sensor noise suppression, post-sensor image restoration, optical aberration correction, Wiener filter, chromatic-spatial simulated PSF, pixel-wise PSF interpolation, autoencoder, transformer, deep residual refinement

## Abstract

Image quality in high-resolution digital single-lens reflex (DSLR) systems is degraded by Complementary Metal-Oxide-Semiconductor (CMOS) sensor noise and optical imperfections. Sensor noise becomes pronounced under high-ISO (International Organization for Standardization) settings, while optical aberrations such as blur and chromatic fringing distort the signal. Optical and sensor-level noise are distinct and hard to separate, but prior studies suggest that improving optical fidelity can suppress or mask sensor noise. Upon this understanding, we introduce a framework that utilizes densely interpolated Point Spread Functions (PSFs) to recover high-fidelity images. The process begins by simulating Gaussian-based PSFs as pixel-wise chromatic and spatial distortions derived from real degraded images. These PSFs are then encoded into a latent space to enhance their features and used to generate refined PSFs via similarity-weighted interpolation at each target position. The interpolated PSFs are applied through Wiener filtering, followed by residual correction, to restore images with improved structural fidelity and perceptual quality. We compare our method—based on pixel-wise, physical correction, and densely interpolated PSF at pre-processing—with post-processing networks, including deformable convolutional neural networks (CNNs) that enhance image quality without modeling degradation. Evaluations on DIV2K and RealSR-V3 confirm that our strategy not only enhances structural restoration but also more effectively suppresses sensor-induced artifacts, demonstrating the benefit of explicit physical priors for perceptual fidelity.

## 1. Introduction

High-resolution image sensors now extend beyond digital single-lens reflex (DSLR) cameras to smartphones and drones. This resolution increase reveals optical aberrations and sensor noise, diminishing image quality. Lens imperfections, including spherical aberration, chromatic dispersion, and misalignment errors, combined with high-ISO (International Organization for Standardization) sensor noise, degrade image sharpness and color fidelity.

Image quality depends not only on optical characteristics but also on sensor-level noise. As demonstrated by Healey and Kondepudy (1994) [1], sensor noise exhibits strong signal dependency, where photon shot noise dominates in high-signal regions while read noise and dark current become significant in low-signal areas. Foi et al. (2008) [2] further established that sensor noise follows heteroscedastic Gaussian distributions with intensity-dependent parameters. This signal-dependent noise becomes particularly problematic when combined with optical aberrations, as areas with reduced light transmission due to aberrations experience lower signal-to-noise ratios. El Gamal and Eltoukhy (2005) [3] showed that Complementary Metal-Oxide-Semiconductor (CMOS) sensor quantum efficiency interacts directly with light distribution patterns affected by lens aberrations, suggesting that optical correction can improve sensor utilization efficiency.

The cascading effects of optical aberrations through modern image processing pipelines compound these issues. Zhang et al. (2011) [4] demonstrated that demosaicing algorithms struggle with optically degraded inputs, generating additional artifacts. Similarly, Tian and Chen (2012) [5] found that poor optical quality necessitates aggressive sharpening, introducing new artifacts. These cumulative degradations underscore the need for addressing optical aberrations as a foundational step in improving overall image quality.

Sensor noise and optical aberrations arise from different sources, yet our research confirms that Point Spread Function (PSF) correction produces cleaner outputs without explicit noise modeling. This aligns with Hasinoff et al. (2010) [6], who established the theoretical relationship between optical efficiency and signal-to-noise ratio in digital imaging systems. By correcting optical aberrations, we improve photon collection efficiency, indirectly enhancing the signal component of the signal-to-noise equation.

To recover the original scene, we must reverse this process using knowledge of the optical blur—often modeled as a PSF. A widely used solution is the Wiener filter, which assumes that the observed image is the result of convolving the original image with a PSF and adding noise. The Wiener filter aims to suppress noise while recovering sharp details, making it an optimal choice under this model. However, it relies on an accurate PSF estimate and moderate noise levels to perform well. To support this step, we first estimate a physically meaningful PSF map and then apply the Wiener filter to restore the image.

In optics, PSF describes how light spreads across sensors through lenses. While ideal systems maintain uniform PSFs, real lenses create position-dependent variations in PSF shape, size, and orientation. These variations resist exact mathematical formulation, necessitating approximation methods. Gaussian-based PSF models, though not an exact physical model, are widely used in computational imaging due to their computational simplicity and their practical approximation of local optical blur.

Pixel-level distortion simulation captures optical aberration changes that are difficult to capture in patch-based methods. Instead of directly modifying image pixels, and assuming uniform patch characteristics, we use parametric approximations of blur intensity, color variation, and orientation characteristics to generate physically simulated PSFs that reflect both chromatic and spatial variations based on adjacent structural features.

Since images are formed by convolution with spatially varying PSFs, direct PSF interpolation better preserves optical properties. This method compensates for color and focus errors and better preserves the structure of the image even in the presence of strong distortion.

In this paper, we propose an interpolation method using pixel-wise chromatic-spatial simulated PSFs, guided by feature similarities across locations. To simulate these PSFs, we apply a simple circular blur function with increasing standard deviation as the distance from the image center increases, simulating field-dependent blur. Chromatic shifts between red, green, and blue channels are also introduced according to lens curvature, reflecting axial dispersion. These simulated PSFs preserve typical optical distortions found in consumer lenses and are used to guide interpolation at each location.

After generating a PSF at each target location, we use a deep learning method to generate a PSF that better reflects local features in a high-dimensional latent space, measure how similar it is to four adjacent PSFs at the required point, and combine them into a new interpolated PSF, weighted by similarity. This results in a PSF that better reflects local features and is more accurate than using basic interpolation alone. Interpolation by the generated PSFs more accurately captures optical aberrations than conventional patch-based or region-based blur models and maintains physical consistency better than pixel-level reconstruction methods. In this approach, the process precisely estimates the PSF characteristics at each target location.

Previous studies such as Mildenhall et al. (2018) [7] and Sitzmann et al. (2018) [8] explored joint optimization of optical and digital components, but these methods required specific models for each lens configuration. Similarly, Li et al. (2021) [9] and Chen et al. (2021) [10] proposed methods relying on pre-computed PSFs—using calibration or simulation—achieving high performance, yet they remained constrained by lens-specific setups and computational complexity. In contrast, our framework requires no neural network retraining for different lenses, achieving adaptability through PSF adjustment alone. This accommodates optical system variations across lens types and wavelength ranges, addressing a key limitation in existing approaches.

We validate our approach using the DIV2K synthetic dataset and RealSR Canon2-LR 2× real-world dataset. These test environments isolate optical correction effects and demonstrate performance under realistic conditions. For benchmark comparison, we adopted previous methods [9,10] adapted for our setting, where explicit lens models or optical prescriptions are unavailable. Compared to Deformable ResU-Net (Chen et al. (2021) [10]), which focuses on optical aberration correction in postprocessing, our preprocessing-based approach showed superior performance. Quantitative results demonstrate +2.7 dB PSNR, +0.039 SSIM, and −0.0535 LPIPS improvements on DIV2K and +1.1 dB PSNR, +0.019 SSIM, and −0.0428 LPIPS on RealSR, confirming that our optical correction approach enhances both signal quality and perceptual image fidelity.

As a result, in addition to optical distortion, it improves control over a variety of factors, including photon shot noise, contrast loss due to stray light, dynamic range suppression, and sharpening artifacts, which are advantageous for sensor-level signal preservation in optically degraded environments.

Our proposed PSF Interpolation Aberration Correction (PIABC) framework comprises:

- Dense PSF representation interpolation using a shallow transformer;
- Wiener filtering with the interpolated PSFs;
- Spatial signal refinement through a deep residual U-Net.

Our transformer architecture features:

- Dense PSF estimation through pixel-level PSF embedding;
- Autoencoder (AE) latent space PSF interpolation using learned representations;
- Cross-attention-based PSF refinement between patches;
- Long-range dependency capture ensuring global correction coherence.

The PIABC framework demonstrates consistent performance across optical systems, enabling accurate image restoration under complex aberrations through adaptive pixel-level PSF interpolation. Unlike previous approaches that address optical aberrations in isolation from sensor characteristics, our method implicitly improves overall signal quality, thereby reducing the impact of sensor noise without direct noise modeling.

## 2. Relative Works

Recent developments in computational imaging have produced a wide range of aberration correction strategies. We classify and summarize the most relevant approaches below.

### 2.1. Interrelation Between Optical Aberration Correction and System-Level Artifacts

Optical aberrations not only reduce the sharpness and color accuracy of raw images but also introduce challenges for downstream image processing. Studies have shown that algorithms such as demosaicing, denoising, and sharpening are sensitive to optical quality, and their performance deteriorates when the input is degraded by aberrations. Zhang et al. (2011) [11] highlighted that demosaicing under aberrated conditions amplifies color artifacts, while Tian and Chen (2012) [5] observed that aggressive sharpening in poorly focused images introduces ringing and aliasing. These effects accumulate and lower overall image quality, especially in compact devices where processing pipelines are highly compressed.

Heide et al. (2014) [12] proposed a unified processing pipeline, FlexISP, to jointly model optical and digital components, demonstrating that optimizing the optics-processing combination yields superior results. Schuler et al. (2016) [13] and Shih et al. (2012) [14] further emphasized that poor optical inputs constrain the effectiveness of image restoration algorithms. These findings suggest that addressing aberrations at the source not only improves native image quality but also mitigates secondary distortions introduced during enhancement steps.

### 2.2. Correction of Sensor-Induced Degradation and Optical Aberrations

Sensor noise and optical aberrations are the two principal sources of image degradation in modern digital imaging systems. Electronic noise—including thermal, read, and quantization noise—interacts with lens-induced blur, producing spatially heterogeneous distortions across the sensor plane. This interaction becomes more pronounced in low-light scenes or with high-ISO (International Organization for Standardization) settings (Kondepudy, 1994 [1]; Foi et al., 2008 [2]). As CMOS (complementary metal-oxide-semiconductor) quantum efficiency and photon collection are directly affected by optical transmission (El Gamal and Eltoukhy, 2005 [3]), mitigating lens aberrations improves sensor utilization efficiency and reduces signal-dependent noise effects.

These insights motivate the integration of optical modeling into image correction frameworks, especially in mobile or low-cost sensors where hardware limitations amplify these interactions. In this context, we adopt a simulation-based approach that approximates chromatic and spatial blur using parametric Gaussian point spread functions (PSFs). Our model uses radial functions in polar coordinates to replicate the smooth variation of blur and color shifts, reflecting common patterns in consumer-grade optics. This method avoids the need for lens-specific calibration and remains compatible with real-world degraded input.

The simulated PSFs are embedded in a latent space via a lightweight autoencoder (AE), enabling structured and continuous interpolation. These PSFs are then applied through Wiener filtering, followed by residual correction using residual U-Net. By modeling degradation before correction, our approach addresses both optical and sensor-level artifacts at the source, improving structural fidelity and perceptual quality across diverse conditions.

### 2.3. Comparison with Existing PSF Modeling Approaches

Aberration correction methods can be broadly categorized into analytical PSF modeling based on physical optics, patch-based statistical correction, and learning-based interpolation frameworks.

Classical approaches derive PSFs using Zernike or Seidel expansions under ideal or calibrated wavefront assumptions (Goodman, 2017 [15]). While interpretable, they require precise calibration and are typically limited to axis-symmetric distortions. Kee et al. (2011) [16], for instance, proposed a parametric blur model fitted from calibration images, expressing PSF variation as a function of spatial coordinates and lens parameters. Although accurate, such methods require dense, lens-specific calibration and generalize poorly to mobile or unknown systems. Wavefront-based approaches often employ Zernike polynomials to represent wavefront errors (Stallinga, 2010 [17]; Siddik et al., 2023 [18]), providing a physically accurate description but at the cost of high computational complexity and calibration requirements.

In contrast, PIABC approximates spatial and chromatic PSF variation using radial functions in polar coordinates, avoiding calibration while maintaining interpretability.

Patch-based methods, such as non-blind deconvolution (Zhang, 2019 [19]) or kernel prediction (Mildenhall et al., 2018 [7]), estimate local blur from image statistics but ignore global optical structure, often treating patches independently.

Recent learning-based approaches embed PSFs into latent spaces. Jing et al. (2021) [20] employed rank-minimizing autoencoders for structured blur representation, and Chen et al. (2021) [10] used raytraced wavefronts to simulate spatially varying PSFs with chromatic shifts, achieving 44.77 PSNR on DSLR data. Monakhova et al. (2020) [21] applied deep models with simulated PSFs for tomographic reconstruction but were limited to static setups.

Gaussian-based PSF models are widely used in computational imaging frameworks due to their computational simplicity and tractability (Chouzenoux et al., 2019 [22]). While efficient, Gaussian-based PSFs may not fully capture higher-order off-axis aberrations or complex wavefront structures present in real lenses.

PIABC initially employs isotropic Gaussian PSFs with radially varying blur and chromatic displacement, capturing realistic RGB shift patterns (e.g., r < g < b) observed in consumer optics. This isotropic baseline is chosen for its computational simplicity before introducing anisotropic Gaussian PSFs at the interpolation stage. These are interpolated through a transformer-based attention mechanism and refined by residual learning. Unlike prior methods, PIABC models PSF at the pixel level without calibration, bridging interpretable physics and scalable restoration while remaining robust to sensor-level noise variations.

## 3. Methods

**Figure 1:**
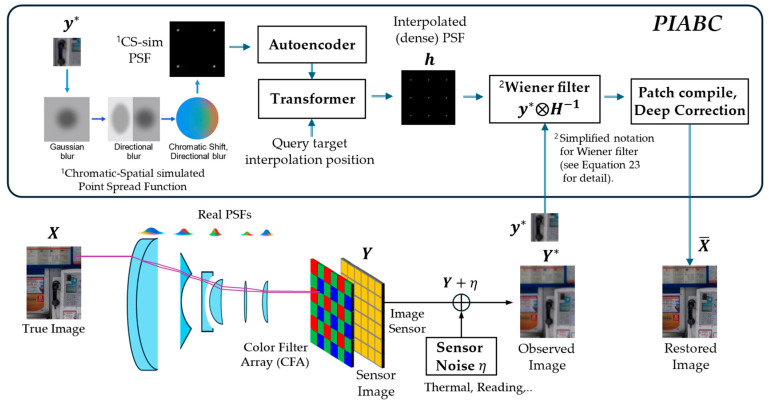
Proposed end-to-end pipeline for sensor-based image restoration: The unboxed area illustrates the conventional image acquisition process, where unknown blur (PSF, point spread function) and sensor-induced electrical noise degrade image quality. Improving optical quality often leads to simultaneous enhancements in sensor performance, such as increased efficiency, reduced photon shot noise, and an extended dynamic range. In the proposed PIABC framework, spatially varying PSFs are first estimated from degraded sensor signals using a transformer-based interpolation method. These PSFs are then applied via Wiener filtering and further refined through residual correction to restore high-frequency details. The entire framework provides interpretable correction for optical aberrations and also demonstrates that, as a secondary benefit, optical restoration can reduce sensor-related artifacts. Here, η is sensor noise, and $y^{*}$ and h denote the patch of the degraded observation $Y^{*}=Y+\eta$ and the corresponding patch of the interpolated PSF field H respectively.

We conceptually illustrate the overall framework in Figure 1, highlighting that our transformer-based interpolation module uses only the autoencoder (AE) embedding of sub-patch point spread functions (PSFs) as input. Notably, image sub-patches are not used in the transformer but are directly processed via Wiener filtering. This preserves the modular design and allows the transformer to focus exclusively on modeling spatially varying PSFs without interference from image content. However, this design choice also limits the transformer’s ability to capture interactions between the PSF and image content.

**Figure 2:**
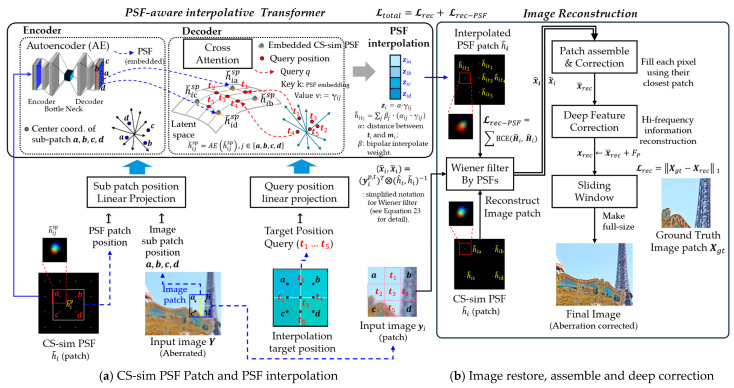
Concept of PIABC. (**a**) CS-sim PSF Patch (initial and interpolated): Each image patch is subdivided into four regions, from which CS-sim PSFs are embedded into a latent space using a trained autoencoder. A transformer decoder computes attention between a query position (marked with an arrow) and these sub-patches to interpolate the corresponding PSF. Query position ***t****_i_* is provided independently as input and serves as the query in the transformer cross-attention. PSF interpolation block: The attention output ($\gamma_{ij}$) from the cross-attention is combined with position similarity weight ($\alpha_{ij})$ to produce interpolated PSF vectors ($z_{ij}^{a,b,c,d})$, shown vertically stacked in the figure. (**b**) Image restoration, patch assembly, and deep correction: interpolated PSFs are used sequentially for Wiener deconvolution and residual correction to reconstruct the final restored image. All steps are described in detail in Section 3.2, Section 3.3 and Section 3.4. For the overall query and correction structure, see Figure 3; for details of the transformer architecture, see Figure 4, and for the residual correction pipeline, see Figure 5. Note: Arrows are shown as examples, and only two are depicted here for clarity.

The proposed model is described in the following stages, and the overall framework is illustrated in Figure 2:

- Formulation;
- Generating chromatic-spatial simulated PSF (CS-sim PSF);
- PSF interpolation on latent space using AE and vision transformer (ViT);
- Patch-wise image restoration using a Wiener filter;
- Patch assembling and full-image compilation;
- Deep residual correction using a residual U-Net (Res U-Net);
- Training objects and loss functions.

### 3.1. Formulation

The primary challenge in aberration correction is estimating the PSF at every point in the lens field—an expensive process if done via direct physical computation. As discussed in Section 2.3, autoencoders can learn a latent space that supports interpolation between measured or simulated PSFs (Jia et al. [23]), reducing reliance on system-specific calibration. Inspired by that data-driven perspective, we propose not to compute the exact physical function at position p but rather to approximate it with an encoder–decoder framework, refined through interpolation. We denote vectors in boldface and scalars in regular font. This convention is applied consistently throughout all interpolation and PSF modeling steps.

Under this assumption system (Schuler et al. [24]), aberration correction can be formulated as an inverse problem, where the goal is to recover the original image $x\left(\cdot \right)$ from its degraded observation $y\left(p\right)$ at position p, considering contributions from other pixel position $p^{\prime }\in P$ that influence the observed degradation:

(1) $$y\left(p,c\right)=\sum_{p^{\prime }\in P}x\left(p^{\prime },c\right)⨂h_{p^{\prime },c}\left(p\right)+\eta \left(p,c\right)$$

where $h_{p^{\prime },c}\left(p\right)$ is transfer function at pixel position $p^{\prime }$ to the other pixel position p, c denotes channel or wavelength, and ⨂ represents the convolution operator. The key challenge is that blur kernel $h\left(\cdot \right)$ is typically unknown, making the problem ill-posed. Non-blind methods assume that $h\left(\cdot \right)$ is known and applies it with deconvolution techniques to estimate $x\left(\cdot \right)$, whereas blind methods jointly estimate both $h\left(\cdot \right)$ and $x\left(\cdot \right)$ directly during the reconstruction process (Levin et al. [25], Yue et al. [26], Li et al. [27]). Let H be the true optical PSF, and $f_{aber}(\hat{p})$ the estimated PSF at the interpolated location $\hat{p}$ of observed locations ${\left\{p_{j}^{*}\right\}}_{j=1}^{m}$, using interpolation weights $\alpha_{j}$:

(2) $$\hat{p}=\sum_{j=1}^{n}\alpha_{j}\cdot p_{j}^{*},\mathrm{where}\;\sum_{j=1}^{n}\alpha_{j}=1,\alpha_{j}\in R$$

Given this formulation, we approximate the $H(\hat{p})$ as

(3a) $$H(\hat{p})\approx f_{aber}\left(\hat{p};{\left\{p_{j}^{*}\right\}}_{j=1}^{m},\varphi ,c\right)$$

(3b) $$=f_{dec}\left(f_{enc}\left({\tilde{H}}_{\varphi }\left(\hat{p}\right)\right);{\left\{p_{j}^{*}\right\}}_{j=1}^{m},\varphi ,c\right)$$

(3c) $$\approx f_{dec}\left({\hat{f}}_{enc}\left(\hat{p},f_{enc}\left({\left\{{\tilde{H}}_{\varphi }\left(p_{j}^{*},c\right)\right\}}_{j=1}^{m}\right)\right)\right)$$

(3d) $$=f_{dec}\left(\sum_{j=1}^{m}f_{sim}\left(p_{j}^{*},\hat{p};\varphi ,c\right)\cdot f_{enc}\left({\tilde{H}}_{\varphi }\left(p_{j}^{*},c\right)\right)\right)$$

where m is the number of nearest observed positions; φ is the aberration such as distortion, chromatic, coma, etc.; ${\tilde{H}}_{\varphi }\left(\cdot \right)$ is the initial CS-sim PSF for transformer input (Section 3.2); $f_{enc}\left(\cdot \right)$ is the encoder function; $f_{dec}\left(\cdot \right)$ is the decoder function; and $f_{sim}\left(\cdot \right)$ is the position similarity function.

Exploiting the natural smoothness of PSF variations between nearby spatial positions, we approximate unmeasured PSFs through weighted interpolation in the autoencoder’s latent space, avoiding the complexity of exact physical wavefront modeling.

The first approximation of Equation (3a) indicates that the proposed PSF depends on PSF patch interpolation, wavelength, and aberration as functions. Equation (3b) replaces direct computation of the physical PSF, including aberration φ at position p, with an encoder–decoder model embedded in latent space. This approximation ensures that the model output aligns with the actual PSF characteristics. The second approximation in Equation (3c) assumes interpolation within the latent space. While the encoder and decoder contain non-linear activation functions and attention weights, PSF variations between adjacent pixels can still be approximated using weighted sums in the decoder in (3d). We incorporate Equations (3b)–(3d) with a transformer to manage the positional relationships and patch similarities, yielding a flexible architecture capable of capturing complex aberrations (including chromatic and rotational factors, as outlined in Section 3.2. Subsequent sections detail how these interpolated PSFs feed into our Wiener filter (Section 3.4.1) and eventually a residual U-Net for high-frequency restoration (Section 3.4.3).

### 3.2. Generating Chromatic-Spatail Simulated PSF (CS-sim PSF)

In this section, we formulate a pixel-wise blur simulation that approximates both geometric and chromatic aberration effects observed in lens-based imaging systems. Rather than relying on physical calibration or optical measurements, we analytically construct spatially varying PSFs using parameterized Gaussian kernels. We refer to the Gaussian-based PSF model in Chouzenoux et al. (2019) [22] as a basis and apply it with pixel-wise chromatic and spatial variation in our transformer-based interpolation framework.

For each pixel p with center coordinate $(o_{x},o_{y}$), we define the reference isotropic Gaussian kernel (representing baseline blur) as:

(4) $$G_{out,c}\left(x,y;x_{0},y_{0},\sigma_{out,c}\right)=\frac{1}{2\pi \sigma_{out,c}^{2}}\exp\left(\frac{{\left(x^{\prime }-x_{0}\right)}^{2}+{\left(y^{\prime }-y_{0}\right)}^{2}}{2\pi \sigma_{out,c}^{2}}\right),$$

where $G_{out,c}\in R^{H\times W}$, $c\in \left\{R,G,B\right\}$ is the channel; $\sigma_{out,c}$ denotes the outer (global) deviations, respectively; and ($x_{0},\;y_{0})$ is the center coordinate.

To introduce anisotropy and directional blur, we define the elliptical Gaussian kernel with chromatic and spatial distortion:

(5) $$G_{in,c}\left(x,y;x_{0},y_{0},\sigma_{in,c},\tau_{c},\rho_{c}\right)=\frac{1}{2\pi \sigma_{in,c}^{2}}\exp\left({-\Delta }^{T}\Sigma_{in,c}\Delta \right),$$

(6) $$\Sigma_{in,c}=R\left(\theta \right)\cdot \left[\begin{array}{cc} \rho_{c}\sigma_{in,c} & 0\\ 0 & \rho_{c}^{-1}\sigma_{in,c} \end{array}\right]{\cdot R\left(\theta \right)}^{T},\;\theta =arctan\left(\frac{y}{x}\right),$$

(7) $$\Delta ={\left[x-x_{0}-∆c\right]}^{T},\;c\in \left\{R,G,B\right\}$$

(8) $$∆R=k_{R}\cdot f\left(d\right)\cdot n_{x,y},∆G=k_{G}\cdot f\left(d\right)\cdot n_{x,y},∆B=k_{B}\cdot f\left(d\right)\cdot n_{x,y},$$

where ΔR, ΔG,ΔB∈R, are distances of pixels due to chromatic aberration, $k_{R},\;k_{G},k_{B}$ are coefficients of chromatic aberration, and $f\left(d\right)$ is a distance-based transformation function. $n_{x,y}$ denotes the normalized vector from the optical center to location (x,y). $f\left(d\right)$ and $n_{x,y}$ are defined respectively as:

(9) $$f\left(d\right)={\left(\frac{dist}{d_{max}}\right)}^{\gamma }$$

(10) $$n_{x,y}=\frac{{\left[x^{\prime }-x_{0},y^{\prime }-y_{0}\right]}^{T}}{\left\|\left[x^{\prime }-x_{0},y^{\prime }-y_{0}\right]\right\|},$$

where $dist=\sqrt{{\left(x^{\prime }-x_{0}\right)}^{2}+{\left(y^{\prime }-y_{0}\right)}^{2}}$ represents the Euclidean distance from the center, $d_{max}$ Maximum distance, and γ is the decay factor. If we denote the variable blur due to independent changes in position per channel by index naming dynamic to the Gaussian standard deviation, the Gaussian kernel can be rewritten as:

(11) $${PSF}_{RGB}\left(x,y;x_{0},y_{0},\Delta R,\Delta G,\Delta B\right)=\left[\begin{array}{c} G\left(x-x_{0}-\Delta R_{x},y-y_{0}-\Delta R_{y};\Sigma_{R}\right)\\ G\left(x-x_{0}-\Delta G_{x},y-y_{0}-\Delta G_{y};\Sigma_{G}\right)\\ G\left(x-x_{0}-\Delta B_{x},y-y_{0}-\Delta B_{y};\Sigma_{B}\right) \end{array}\right]$$

where ${PSF}_{RGB}(\cdot ,\cdot \;;x_{0},y_{0})\in R^{H\times W\times 3}$, $\Sigma_{c}$ is the standard deviation of each channel. To reflect the fact that the amount of blur increases with distance from the lens center, the Gaussian standard deviation is redefined as follows:

(12) $$\Sigma_{final,\;c}\left(x,y\right)=\sigma_{out,c}\Sigma_{in,c}\cdot \left(1+\alpha_{c}f\left(d\right)\right),$$

where $\Sigma_{final,c}\in R^{H\times W\times (2\times 2)}$, $\alpha_{c}$ is distance-sensitive modulation coefficient.

Finally, by integrating the elements, the initial PSF originated from $\left(x_{0},y_{0}\right)$ is formulated as:

(13) $${\tilde{H}}_{\varphi }\left({\hat{p}}_{i},c\right)={PSF}_{init,c}\left(x,y\right)=W_{c}\cdot G\left(x-x_{0}-\Delta c_{x},y-y_{0}-\Delta c_{y};\Sigma_{final,\;c}\right),$$

where ${PSF}_{init,c}\in R^{H\times W\times (H\times W)}$ denotes the initial PSF generated through a chromatic-spatial PSF simulation process. $W_{c}$ is the weight that modulates the contribution of each color channel (R,G,B) to the final aberration correction, and G is the spatially varying Gaussian function with chromatic displacement Δc.

This process enables pixel-specific variation of the PSF, reflecting how aberration differs at each location and across color channels. It serves as a simplified yet physically interpretable initialization for learning-based PSF interpolation.

### 3.3. Spatially Varying PSF Interpolation for Noise Reduction Using Deep Learning

#### 3.3.1. Query Patch Configuration for Spatially Varying PSF Interpolation

**Figure 3:**
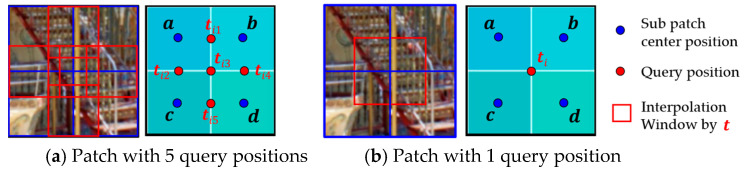
Query configuration for interpolation in Cartesian coordinate system: (**a**) Five query positions $t_{i1}\sim t_{i5}$ are placed at the midpoints between neighboring PSF centers in a 256 × 256 patch, which may cause overlapping windows, depending on query settings; this overlap case is illustrated here for clarity; (**b**) A single query point $t_{i}$ is at the center of a 128 × 128 patch; Subpatch centers and queries are marked by blue and red dots, respectively, and interpolation windows by red boxes.

To support PSF interpolation across spatially varying regions, we design two types of query configurations based on patch size. For 256 × 256 patches, five interpolation points are used between patch centers (see Figure 3a); for 128 × 128 patches, a single central point is used (Figure 3b). These key query points determine the spatial density of interpolated PSFs, enabling locally adaptive aberration correction. A trade-off exists between interpolation precision and computational cost, which we evaluate in Section 4.

#### 3.3.2. PSF Patch Embedding with Autoencoder

To embed each CS-sim PSF patch into a structured and compact latent space, we employ a vanilla autoencoder (AE). Each PSF patch ${\tilde{h}}_{j}^{p}$ is subdivided into sub-patches indexed by j∈{a,b,c,d}. For each sub-patch, a feature embedding:

(14) $${\overset{˘}{h}}_{ij}^{sp}=PatchEmbedding({\tilde{h}}_{ij}^{sp}){\in R}^{768}$$

which flattens the 33 × 33 × 3 sub-patch into a 3267-dimensional vector and then projects it to 768 dimensions.

This embedding preserves the essential PSF structure while enabling subsequent transformer-based interpolation. During AE training, Gaussian noise with $\sigma =\max\left(\sqrt{Var({\overset{˘}{h}}_{ij}^{sp}}\right),\;5\times 10^{-2}$ is added to each embedding to yield a denoising effect.

The decoder head is implemented as a multi-layer perceptron (MLP) consisting of three fully connected layers (768 → 1536 (ReLU) → 1536 (ReLU) → 3267) with intermediate ReLU activations. Specifically, the architecture first expands the latent feature from 768 to 1536, applies ReLU activation, then maintains this hidden dimension through another 1536-unit layer with ReLU activation, and finally projects the feature vector into the target PSF dimension (e.g., 33 × 33 × 3). After decoding, a softplus activation ensures non-negativity, followed by normalization so that the output PSF sums to one, consistent with physical PSF energy constraints. Note: For clarity, the sub-patch index ‘sp’ is omitted in Figure 2 but is explicitly included hereafter for consistency.

#### 3.3.3. Transformer-Based PSF Interpolation for Enhanced Accuracy

We adopt a vision transformer (ViT) architecture consisting of an encoder and a decoder to perform spatial interpolation of PSFs while maintaining physical consistency.

**Figure 4:**
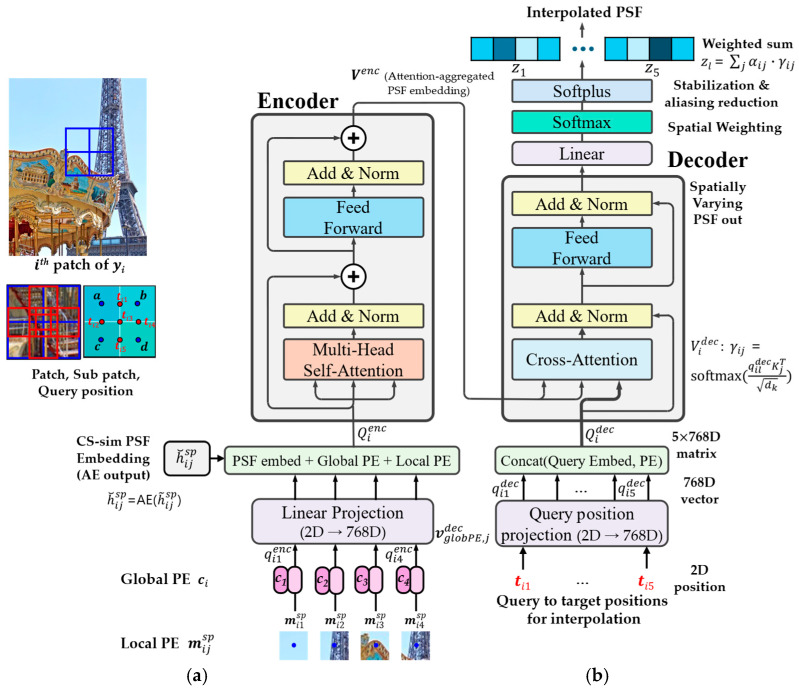
Overview of the proposed PSF-aware transformer architecture: (**a**) Encoder: Each PSF patch is linearly embedded and combined with positional encodings (local and global) to form attention tokens. Attention is computed across sub-patches to capture spatial relationships. AE: Autoencoder. (**b**) Decoder: Queries at target positions are projected into the embedding space and attend to encoder features. In the decoder, the query at position i attends to encoder sub-patches at positions j, and the attention weights $\gamma_{ij}$ determine their relative contributions to the aggregated latent feature $z_{i}$. Attention-weighted aggregation yields latent PSF representations, which are linearly transformed to generate spatially interpolated PSFs. Query configuration follows Figure 3a. Sub-patch index ‘sp’ is omitted in Figure 2 for clarity but included here for detail. Layer Normalization (LN) is applied internally within the encoder. Decoder queries do not directly incorporate Local PE; instead, Global PE is indirectly propagated via attention; Case (a) in Figure 2 is used to illustrate the configuration. The bluish shaded blocks in the upper-right indicate subpatch-wise contributions to the interpolated result, based on the composite weight $\alpha_{ij}\cdot \gamma_{ij}$ where $\alpha_{ij}$ denotes position similarity and $\gamma_{ij}$ is the attention weight learned by the transformer; Relevant positional encodings and notations are indicated by shade intensity in the figure and fully defined in Table 1.

Unlike typical ViTs that use image patches as inputs, our method feeds only the AE-embedded PSF sub-patches to the encoder, focusing the transformer solely on PSF modeling. Each encoder feature vector is constructed by combining the AE embedding of a sub-patch PSF with local and global position encodings. (see Figure 4 and Table 1)

- Encoder: Applies self-attention to aggregated PSF embeddings with positional encodings. Each sub-patch PSF embedding ${\overset{˘}{h}}_{ij}^{sp}$ is combined with local position embedding $PE\left(m_{j}^{sp}\right)$ and global position embedding $GlobalPE\left(c_{i}\right)$. The encoder input query is then formulated as:
  (15) $$Q_{j}^{enc}={\overset{˘}{h}}_{ij}^{sp}+LinearProjection(Local\;PE\left(m_{j}^{sp}\right)+GlobalPE(c_{i})$$
  where j indexes sub-patches within the PSF patch i. Although layer normalization (LN) is not explicitly shown here, it is applied internally within the transformer encoder block. This step preserves both local and global spatial context in the encoder tokens. $m_{j}^{sp}{\in R}^{2}$ denotes the center coordinates of each sub-patch.
- Decoder: For each target query position $t_{il}{\in R}^{2}$, the decoder query vector vector $q_{il}^{dec}$ is computed as:
  (16) $$q_{i,l}^{dec}=LinearProjection\left(t_{il}\right)+GlobalPE(c_{i}),$$
  This decoder query interacts with the encoder outputs via a cross-attention block, producing attention weights $\gamma_{ij}$ and aggregated latent features ${\overset{˘}{h}}_{ij}^{sp}$:
  (17) $$\gamma_{ij}=softmax\left(\frac{q_{i,l}^{dec}\cdot k_{j}^{T}}{\sqrt{d_{k}}}\right)$$
  where $k_{j}$ represents the encoder key vector, and $d_{k}$ is the key dimension. This mechanism captures the contribution of each sub-patch embedding to the interpolation target.
- Position Similarity: Position similarity between the query position and each sub-patch center is defined as:
  (18) $$\alpha_{ij}=softmax(-{\left\|{t_{i,l}-m}_{ij}^{sp}\right\|}^{2}),$$
  where j∈{a,b,c,d} indexes the neighboring sub-patches. This term quantifies the geometric proximity between the interpolation target and each sub-patch center.
- Latent Aggregation: The position similarity and attention weights are combined to produce the weighted latent feature:
  (19) $$z_{ij}=\alpha_{ij}\cdot \gamma_{ij},$$
- Interpolation Step: The final interpolated PSF at the query position $t_{il}$ is then obtained as:
  (20) $${\hat{h}}_{it_{l}}=\;\sum_{j}\beta_{j}\cdot z_{ij,}$$
  where $\beta_{j}$ denotes the bilinear interpolation weight derived from the relative distances between the target position $t_{il}$ and the sub-patch centers $m_{ij}^{sp}$:
  (21) $$\beta_{j}=\prod_{d\in \{x,y\}}\left(1-\left|{t_{i}^{d}-m}_{ij}^{d}\right|\right),$$
  Finally, a linear projection is applied to $z_{i}$ to generate the interpolated PSF $\hat{H}$:
  (22) $${\hat{H}}_{i}=Linear(z_{i})$$

This step aggregates sub-patch contributions into a single PSF at location $t_{il}$. This formulation decouples spatial attention (via $\gamma_{ij}$) from position similarity (via $\alpha_{ij}$), allowing stable and structured interpolation without relying on raw coordinate distances.

Table 1 summarizes the stages and notations involved in the transformer-based PSF interpolation pipeline. Importantly, at the interpolation stage, the query index l is aggregated through weighted sums into the final position index i (see Figure 2). This notation aligns with Table 1 and ensures seamless integration between encoder–decoder outputs.

### 3.4. Image Restoration Using Image Restoration Using Wiener Filtering and Deep Correction

**Figure 5:**
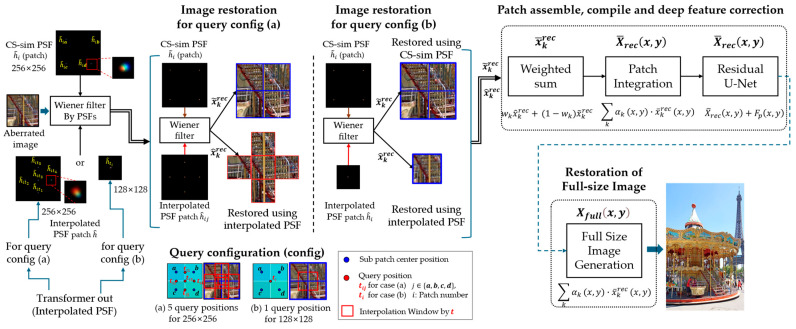
Patch assembly and compilation pipeline using Wiener Filter image restoration: The left part illustrates the input aberrated image patches and the interpolated PSFs generated by the Transformer for each query configuration (see Figure 3). The center part shows how the interpolated PSFs are combined with the Wiener filter to reconstruct image patches under each setting. The right part depicts α-weighted fusion of reconstructed results from CS-sim and interpolated PSFs, followed by patch integration, deep feature correction using a residual U-Net (see Section 3.4.3), and final full-size image restoration.

For high-resolution image restoration, we employ a Wiener filter for frequency-domain restoration, followed by patch assembly and compilation. This integrated approach, which will be depicted in Figure 5, ensures the effective enhancement of fine details. As will be discussed in Section 3.4.3, ResU-Net [28] further improves high-frequency detail recovery.

#### 3.4.1. Patch Image Restoration Using Wiener Filter

While the Wiener filter is widely used for frequency-domain restoration, it often introduces ringing artifacts near sharp edges. To address this, we employ PSFs interpolated by the transformer model, which incorporate spatial variations and suppress artifacts during high-frequency restoration. The Wiener filter-based image restoration process for a patch *k* at location p is formulated as follows:

(23) $${\hat{X}}_{k}\left(u\right)=\frac{H_{k,c}^{*}\left(u\right)}{{\left|H_{k,c}\left(u\right)\right|}^{2}+K_{k,c}}\cdot Y_{k}\left(u\right),$$

where $Y_{k}\left(u\right)$ is the observed patch, $H_{k,c}\left(u\right)$ is the PSF in the frequency domain, $K_{k,c}$ is a constant reflecting signal-to-noise characteristics, and $H_{k,c}^{*}\left(\cdot \right)$ is the complex conjugate frequency representation of the interpolated PSF. The result ${\hat{X}}_{k}\left(u\right)$ is transformed back to the spatial domain as:

(24) $$x_{k}^{rec}\left(u\right){=F}^{-1}\left\{{\hat{X}}_{k}\left(u\right)\right\}$$

This approach allows local variations in PSFs to be incorporated into the restoration process, reducing artifacts compared to global Wiener filtering. Consequently,

(25a) $${\hat{x}}_{k}^{rec}{=F}^{-1}\left\{\frac{{\hat{H}}_{i}^{*}}{{\left|{\hat{H}}_{i}\right|}^{2}+K}F\left(P_{k}^{\hat{H}}\right)\right\},$$

(25b) $${\tilde{x}}_{k}^{rec}{=F}^{-1}\left\{\frac{{\tilde{H}}_{i}^{*}}{{\left|{\tilde{H}}_{i}\right|}^{2}+K}F\left(P_{k}^{\tilde{H}}\right)\right\},$$

where, $P_{k}^{\hat{H}}$ and $P_{k}^{\tilde{H}}$ represent the patch domains used for each Wiener filtering step, combining the main support region $P\left(a,\;b,\;c,\;d\right)$ and the corresponding PSF query positions $P_{\tilde{H}}$. The terms ${\hat{x}}_{k}^{rec}$ and ${\tilde{x}}_{k}^{rec}$ denote the restored patches using PSFs ${\hat{H}}_{i}$ and ${\tilde{H}}_{i}$ respectively, where ${\hat{H}}_{i}^{*}$ and ${\tilde{H}}_{i}^{*}$ are conjugate frequency-domain representations. $F\left(\cdot \right)$ and $F^{-1}\left(\cdot \right)$ denote the Fourier transform and its inverse, respectively, and *K* is the noise control factor.

In Equations (23)–(25b), the scalar parameter K represents the ratio between the noise and signal power spectral density, i.e., $K=\sigma_{noise}^{2}/\sigma_{signal}^{2}$ which controls the trade-off between deblurring and noise amplification in Wiener filtering. A smaller K preserves fine details at the risk of amplifying noise, while a larger K suppresses noise more aggressively but may over-smooth image features.

In real-world deployments, noise characteristics vary significantly across optical designs, sensor types, and lighting conditions. Using a fixed K avoids the complexity and potential instability of dynamic noise estimation while enabling consistent and computationally efficient preprocessing.

Moreover, modern architectures such as the residual U-Net demonstrate strong robustness to fixed preprocessing patterns. Through their hierarchical feature representation learning, they effectively compensate for suboptimal filtering artifacts, making the precise tuning of K less critical for overall restoration performance. This empirical value is later validated through dataset-wide sensitivity analysis (see Appendix A).

#### 3.4.2. Patch Assembly Using Transformer-Interpolated PSFs

Figure 5 illustrates this process, summarizing the overall patch assembly and correction pipeline.

Once the patches are restored using the Wiener filter, they are combined to form a full-resolution image using a learned weighting strategy. The filtered outputs based on different PSF configurations are first merged as:

(26) $${\bar{x}}_{k}^{rec}=w_{k}{\hat{x}}_{k}^{rec}+\left(1-w_{k}\right){\tilde{x}}_{k}^{rec},$$

where $w_{k}$ is a learned weight that balances the contributions of the two filtering results.

To assemble the full image from overlapping patches, the restored patches are spatially blended using attention-based weighting:

(27) $${\bar{X}}_{rec}\left(x,y\right)=\sum_{k}\gamma_{k}\left(x,y\right)\cdot {\bar{x}}_{k}^{rec}\left(m,n\right),$$

where $\gamma_{k}\left(x,y\right)$ s the transformer-derived spatial attention weight that determines each patch’s contribution, normalized as:

(28) $$\sum_{k}\gamma_{k}\left(x,y\right)=1,$$

which determines the contribution of each patch to the final restored image. To stabilize this weighting process, we apply a softplus activation to the attention output before normalization:

(29) $$z_{i}=softplus\left(V_{i}^{dec}\right),$$

where $V_{i}^{dec}$ is the decoder output from the transformer, as described in Table 1.

To further ensure seamless transitions between patches, a boundary blending mask is applied:

(30) $${\bar{X}}_{rec}\left(x,y\right)={\bar{X}}_{rec}\left(x,y\right)\cdot M\left(x,y\right)+{\bar{X}}_{border}\left(x,y\right)\cdot \left(1-M\left(x,y\right)\right)$$

with $M\left(x,y\right)$ defined as a normalized blending mask:

(31) $$M\left(x,y\right)=\frac{\sum_{k}\gamma_{k}\left(x,y\right)}{\sum_{k}\gamma_{k}\left(x,y\right)+\epsilon },$$

where ϵ is a small numerical constant to prevent division by zero. Finally, the Gaussian weighting function used in the mask is given by:

(32) $$G\left(x,y\right)=\frac{exp\left(-\frac{{\left(x-x_{0}\right)}^{2}+{\left(y-y_{0}\right)}^{2}}{2\sigma^{2}}\right)}{\sum_{\left(x^{\prime },y^{\prime }\right)}exp\left(-\frac{{\left(x-x_{0}\right)}^{2}+{\left(y-y_{0}\right)}^{2}}{2\sigma^{2}}\right)}$$

where ($x_{0},\;y_{0})$ represents the center of each patch, and σ controls the smoothness of the blending transition.

The sliding window approach ensures that overlapping regions are blended smoothly, avoiding discontinuities and improving spatial coherence. This step enables deep-corrected patches to be integrated into a fully restored, high-quality image.

#### 3.4.3. Deep Feature Correction Using Residual U-Net

The initial PSFs $\tilde{H}$ and interpolated PSFs $\hat{H}$ exhibit complementary spatial characteristics: $\tilde{H}$ is locally sharper and exhibits higher peak intensity due to its direct observation, while $\hat{H}$ tends to be spatially broader and smoother owing to latent space-based interpolation. These differences, while beneficial for robustness in patch-wise PSF coverage, result in spectral inconsistency and potential high-frequency attenuation during synthesis and Wiener filtering. Therefore, the combined image may still exhibit blurred fine structures and edge softening.

To address this, we employ residual U-Net [28] to restore lost high-frequency details by learning spatial priors over multiple PSF-corrected patches, as described below. The restored image from the previous steps suffers from high-frequency loss during the PSF synthesis and Wiener filtering processes.

**Figure 6:**
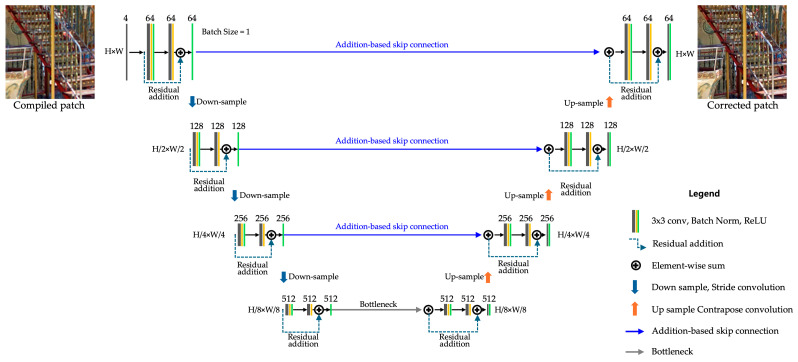
Deep Correction with residual U-Net for our PSF interpolative aberration correction: Input Processing: Fourier-restored image patches of varying sizes are passed through a 3 × 3 convolution filter, converting the input to *B* × 4 × *H* × *W*. Subsequently, it is transformed into 1 × 64 × *H* × *W*; Encoding: Involves three layers of stride convolutions, downsampling the input to a bottleneck layer; Bottleneck: Reduce features to 512 Ch, downsampling by a factor of 1/8 to retain critical information; Decoding: Performed through 3 stages of transpose convolution layers, upsampling the features back to the original dimensions; Residual Blocks: Each encoder and decoder block incorporates two 3 × 3 convolution layers and a batch normalization (BN) layer, followed by element-wise residual addition.

As illustrated in Figure 6, this architecture extends vanilla U-Net [29] by introducing residual connections, improving gradient flow and feature refinement, minimizing computational overhead while effectively restoring fine structures. We implement several optimizations, such as:

- Adaptive Receptive Fields: Given the variation in image dimensions within the DIV2K HR Train [30] and RealSR-V3 [31] datasets, we configured adaptive receptive fields to accommodate patch sizes up to 734 × 734 pixels with 17 × 17 padding.
- Addition-Based Skip Connections: Reduce computational complexity while preserving critical feature information, enhancing segmentation performance in deep learning models such as ResU-Net-a [32] and HRNet (High-Resolution Network) [33].
- Enhanced Decoder with Residual Additions: Residual connections are added within the decoder to improve feature refinement and gradient flow stability.

### 3.5. Training Objects and Loss Functions

It is optimized using two main objectives: PSF interpolation fidelity and final image restoration quality. The total loss is defined as:

(33) $$L_{total}=L_{rec-PSF}+L_{rec},$$

The PSF reconstruction loss $L_{rec-PSF}$ ensures that interpolated PSFs remain consistent with observed PSFs. While BCE loss is commonly used for binary classification, it is well-suited here because our interpolated PSF maps can be interpreted as soft assignment weights normalized between 0 and 1. This facilitates stable training and interpretable interpolation:

(34) $$L_{rec-PSF}=\sum BCE\left({\tilde{H}}_{i},{\hat{H}}_{i}\right),$$

The image restoration loss $L_{rec}$ enforces pixel-wise similarity between the ground truth and restored image using the $l_{1}$-norm:

(35) $$L_{rec}={\left\|X_{gt}-{\bar{X}}_{rec}\right\|}_{1}$$

$L_{total}$ represents the overall optimization objective, $L_{rec-PSF}$ regularizes PSF interpolation, while $L_{rec}$ focuses on perceptual image quality. For models using explicit PSF embeddings, both losses are applied; for non-interpolative variants, only $L_{rec}$ is used.

## 4. Experiments and Results

In this section, we present the experimental results for the point spread function (PSF) Interpolative transformer, restoration filter, and deep restoration methods. We begin by describing the datasets and evaluation metrics, followed by a comparison with benchmark studies. Finally, we discuss ablation studies on parameters and data configurations for the proposed model and evaluate computational overhead.

### 4.1. Experimental Setup

#### 4.1.1. Training Dataset

We employed two datasets for training and evaluation: DIV2K high resolution (DIV2K HR) and RealSR-V3, each serving a distinct experimental purpose.

DIV2K HR was used to evaluate the isolated impact of our simulated PSFs under conditions that minimize sensor influence, enabling validation of the physical consistency and spatial sensitivity of the proposed PSF formulation. The DIV2K dataset contains 800 high-resolution (2K) images, which were degraded using our CS-sim PSF model. This model applies spatially and chromatically varying anisotropic Gaussian blur across the image plane, allowing us to generate diverse aberration patterns without introducing sensor-related artifacts.

RealSR-V3, on the other hand, was employed to validate the model’s generalizability on real sensor-captured images, which inherently include complex and uncontrolled degradations such as chromatic aberration, defocus, geometric distortion, sensor noise, and demosaicing artifacts. We used the Canon2-LR subset, captured with a Canon EOS 5D Mark III (Canon Inc., Tokyo, Japan) and a 24–105 mm f/4.0 zoom lens. Although the dataset provides multiple magnification levels, we selected ×2 scaling, which preserves optical aberration characteristics without introducing aliasing artifacts that commonly occur at higher magnifications.

This Canon2 subset is known to exhibit relatively strong and spatially diverse optical aberrations—particularly in the outer field—due to the zoom lens configuration and wide field of view. These characteristics make it a well-suited benchmark for evaluating pixel-wise PSF estimation and correction under real-world, sensor-integrated conditions.

To maintain consistent lens behavior across both datasets, we applied a common protocol: each distortion case was assigned to a batch of 10 images—5 used for training and 5 for testing—assuming a shared lens profile within each batch.

#### 4.1.2. Training Parameter Configuration

The training process (Equation (28)) was conducted using the hyperparameters based on vanilla transformer [34], vision transformer (ViT) [35], and Lee et al. [36], but with additional modifications to adapt the spatial weighting factor μ that controls how PSF z is applied to nearby query locations by adjusting the attention bias based on spatial distance. The spatial weighting factor (μ) is integrated into the cross-attention computation as:

(36) $$\gamma_{ij}=softmax\left(\frac{{(Q}_{i}W_{Q}){{(K}_{j}W_{K})}^{T}}{\sqrt{d_{k}}}\right)+\mu \cdot P_{ij},$$

where $\gamma_{ij}$ denotes the cross-attention weight between query $Q_{i}$ and key $K_{j}$; $W_{Q}{\;and\;W}_{K}$ are projection matrices; $d_{k}$ is the dimensionality of the key vectors; $P_{ij}$ encodes the relative spatial distance between the query and known PSF positions; and μ∈R is regularized using the softplus function to ensure positivity and numerical stability during training.

As training proceeds, μ is adaptively learned to modulate spatial bias, enabling the model to prioritize local PSF information that is most relevant to the observed image content. This adaptive mechanism improves the ability to characterize fine-scale optical aberrations that vary spatially across the sensor field.

The detailed architectural and training configuration of our transformer module, including optimizer setup, positional encoding, and loss function structure, is summarized in Table 2.

#### 4.1.3. Residual U-Net Configuration

We define the hyperparameters for training Equation (35) based on those used in residual U-Net [28]. To achieve more robust and fine-grained learning, we introduce the following modifications to the loss function:

- Loss Function: $l_{1}$-norm loss;
- Optimizer: Adam optimizer;
- Learning Rate: 0.0001;
- Activation Function: ReLU;
- Batch Size (B): 1.

The proposed approach integrates consistent training and fine-tuning strategies, leveraging stable loss functions and augmentation techniques. These measures ensure robustness across various lens models and distortion conditions.

#### 4.1.4. Chromatic-Spatial Simulated PSF (CS-sim PSF) Setup

We apply our PSF simulation process to both the DIV2K and RealSR-V3 datasets, where PSFs are synthetically generated for training and evaluation.

We simulate the initial PSFs under three different optical conditions across 400 images, considering refractive index variations caused by lens position and wavelength dispersion—two major contributors to high-resolution optical aberrations. Spatially varying aberrations are modeled based on position shifts from the center to the lens edge, while the variation of chromatic aberration with wavelength models the effect of refractive index dispersion in the optical system with wavelength (Section 3.2). To implement these, we design our PSF simulation with the following components:

- Gaussian blur applied uniformly using an isotropic Gaussian function;
- Radial anisotropic transformations to simulate spatially varying distortions;
- Wavelength-dependent distortion modeling (e.g., r<g<b or b<g<r) with varying inner and outer sigma intensities.

In our experiments, lens position variations are applied at 2-pixel intervals from −5 to 15 pixels. Chromatic aberration effects are controlled via outer sigma, which determines global blur intensity, and inner sigma, which modulates local blur based on pixel distance from the center. For the outer sigma, we set it to range from 1.0 to 2.5, while the inner sigma is sampled between 0.8 and 2.0. These parameters create low, medium, and high aberration levels, enabling training under diverse optical conditions.

These simulated aberrations, combined with the diversity and high-resolution conditions of the DIV2K HR dataset, ensure that the generated PSFs closely reflect real-world aberration variation patterns for robust framework validation.

#### 4.1.5. Fine-Tuning Configuration

Unlike the RealSR-V3 dataset, which consists of images captured using a fixed lens configuration (e.g., Canon, Nikon), the DIV2K dataset lacks explicit lens metadata and is synthetically augmented to simulate diverse aberration patterns. To evaluate the model’s adaptability to such variations, we implemented a fine-tuning strategy tailored to DIV2K. Each session consisted of 300 iterations per simulated lens group and was applied to batches of 10 images (5 for training, 5 for testing), assuming shared optical properties. Hyperparameters were consistent with the general training phase (learning rate: 0.0005; step size: 1000). To stabilize training and smooth loss fluctuations, a moving average with a window size of 50 was applied every 100 iterations.

#### 4.1.6. K Parameter Pre-Evaluation for Robust Filtering

We chose a fixed value of K=0.01 based on a preliminary evaluation over a wide range of values [0.0002, 0.1] using the full test sets of DIV2K (*n* = 400) and RealSR-V3 Canon 2 LR (*n* = 50). While this value was not optimal for all scenarios, it showed robust and consistent performance across both synthetic and real-world conditions. Detailed sensitivity analysis is presented in Appendix A.

#### 4.1.7. Computing Environment

All experiments were conducted on a system equipped with an Intel i7 12th Gen CPU, 64 GB RAM, and an Nvidia RTX 3090 GPU with 24 GB VRAM. The experiments were implemented using Python 3.12, PyTorch 2.2.2 (https://pytorch.org) and CUDA 12.2 on Ubuntu 20.04.

### 4.2. Benchmark Test

#### 4.2.1. Performance Metrics

For evaluation, we use SSIM (Structural Similarity), PSNR (Peak Signal-to-Noise Ratio), and LPIPS (Learned Perceptual Image Patch Similarity). SSIM measures the preservation of structural features, indicating whether the restored image retains the original structural details. SSIM assesses whether PSF correction via high-dimensional positional information accurately restores image structures. PSNR evaluates image clarity by quantifying the reduction of blurring and distortion, reflecting how accurately positional information is applied during PSF correction. LPIPS, a perceptual metric based on deep features from pre-trained networks, to assess image similarity from a human-vision perspective. LPIPS has been widely adopted in recent image restoration studies and complements SSIM and PSNR by capturing perceptual quality beyond pixel-level similarity.

#### 4.2.2. Benchmark Models

For methodological comparison, we adapted two hybrid computational optics methods—UABC by Li et al. [9] and Chen et al.’s image simulation framework [10]—to fit our testing environment, maintaining their core principles. Both methods originally used Zemax and lens prescriptions for PSF simulation, unavailable in our setup, so we used synthetic PSFs validated against real aberration patterns and calibrated to mimic real-world distributions (Section 4.4).

- Wiener-Prior UABC-net (adapted from Li et al. [9]): The original UABC uses a PSF-aware deep network, pre-trained on Zemax-simulated PSFs and fine-tuned for specific lenses. We replaced PSF reconstruction with regulated Wiener filtering using synthetic PSFs, keeping the U-Net refinement to reduce artifacts. This preserves the PSF-aware correction focus;
- Patch-based Deformable ResU-net (adapted from Chen et al. [10]): Chen et al. (2021) proposes an imaging simulation system that computes spatially variant PSFs using lens data and raytracing for post-processing, paired with a spatial-adaptive CNN featuring Deformable ResBlocks. We preserve the Deformable ResBlock’s role in handling non-uniform distortions. This ensures the method’s spatial adaptability is retained.

Both adaptations align with our experimental framework while using validated synthetic PSFs to compensate for missing optical priors. While these implementations differ from the original Zemax-based approaches, we mitigated potential discrepancies through empirical validation, acknowledging that our preprocessing-focused PIABC represents a fundamentally different approach to the same optical correction challenge.

#### 4.2.3. Quantitative and Qualitative Comparison on DIV2K Dataset

To evaluate our physically simulated PSFs in isolation from sensor effects, we conducted quantitative and qualitative comparisons on the DIV2K dataset. These high-resolution images were captured under conditions that minimize sensor influence [30], enabling us to highlight the spatial sensitivity and correction accuracy of our method.

Table 3 presents the PSNR and SSIM results for two patch sizes, 256 × 256 and 128 × 128, evaluated under both globally trained and batch-wise fine-tuned conditions. The benchmark includes our proposed method (PIABC) alongside the two adapted baseline models introduced in Section 4.2.2.

Our method outperformed both baselines across all settings. Compared to the fine-tuned patch-based deformable ResU-net, PIABC achieved up to 9.6% higher PSNR and 4.5% improvement in SSIM, showcasing its capacity to generalize spatial information without relying on handcrafted priors. In addition, PIABC achieved an LPIPS score of 0.2036, representing a decrease of 0.0535 compared to the fine-tuned patch-based deformable-net (0.2571), highlighting improved perceptual similarity in the restored images. This performance stems from the use of high-dimensional positional embeddings and shallow self-attention modules within the autoencoder architecture.

Notably, while fine-tuning improved results for most models, PIABC experienced slight performance degradation post-fine-tuning. This suggests that the fine-tuning process, which is tailored to lens-specific distortions, may overfit and inadvertently reduce generalization. Our findings support the conclusion that PIABC’s design benefits more from broad, diverse training data than from batch-specific adaptation. This observation can be further explained by the fact that lens-specific characteristics are already incorporated through the pre-filtered PSFs applied in the Wiener filtering stage, effectively embedding lens-specific information before residual U-Net training. Consequently, additional lens-specific fine-tuning in the residual U-Net does not provide substantial new information but rather risks reinforcing batch-specific features, which can compromise the generalizable latent representations learned during global training.

Due to the patch-based training structure of the residual U-Net and the incorporation of lens-specific priors via Wiener filtering, fine-tuning often fails to produce noticeable fluctuations in validation loss curves or distinct visual artifacts, making such plots less informative for identifying overfitting in our framework. Instead, performance trends were best captured through quantitative metrics, as reflected in Table 3.

**Figure 7:**
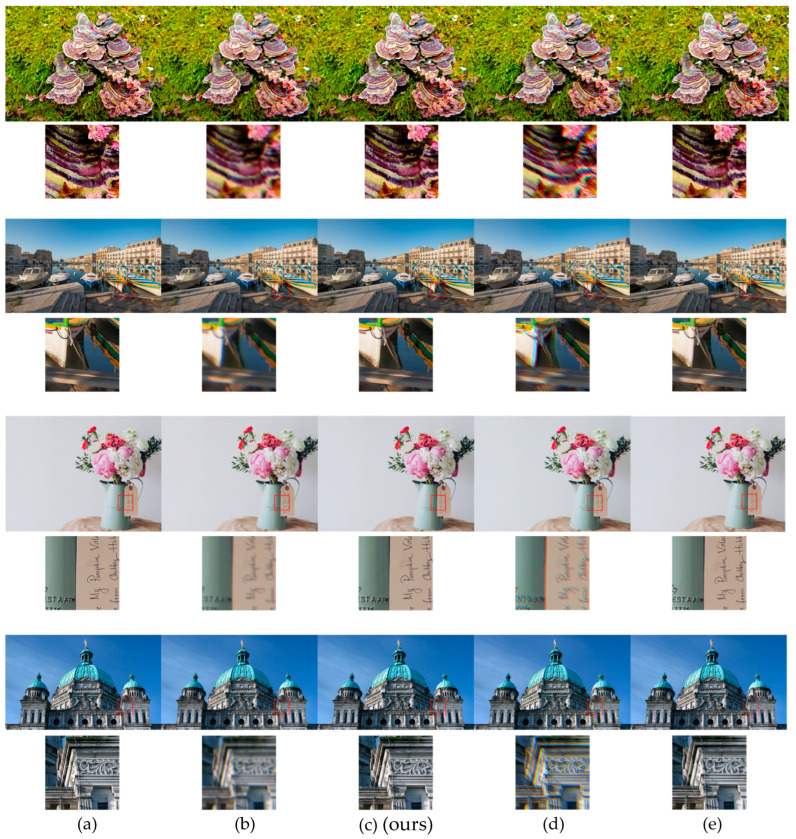
Qualitative comparison of DIV2K dataset: (**a**) ground truth image; (**b**) aberrated image; (**c**) PIABC (Ours, before finetuning); (**d**) Wiener-prior UABC-net (local finetune); (**e**) patch-based deformable-net (local finetune).

Figure 7 illustrates qualitative differences between the models. PIABC successfully corrects chromatic aberration and recovers fine structures, particularly in edge and texture regions. Compared to the baselines, it produces sharper, cleaner reconstructions with better color fidelity.

#### 4.2.4. Quantitative and Qualitative Comparison Using RealSR-V3 Canon 5D3 Dataset

To evaluate the applicability of our method under real-world conditions, we conducted experiments on the RealSR-V3 Canon2-LR dataset, which includes low-resolution images captured using a zoom-lens DSLR camera Canon EOS 5D Mark III (Canon Inc., Tokyo, Japan) under uncontrolled settings. These images contain diverse degradations, such as optical blur, chromatic aberration, and sensor noise, arising from real lenses and imaging hardware [31].

We specifically selected the image Canon_038_LR2.png, which depicts a scene frequently used in prior benchmark works [28], and employed the ×2 magnification version to preserve the spatial variation of optical aberrations. While the RealSR-V3 dataset does not explicitly annotate aberration severity per image, Canon2 images are known to exhibit relatively strong field-dependent distortions due to DSLR lens characteristics. This observation aligns with prior reports that show similar aberration profiles in datasets acquired using low-end mobile and consumer-grade optics (Chen et al., 2021) [10]. PSFs were estimated directly from the LR image using our proposed model and used in subsequent Wiener filtering and deep residual correction stages. This setup allows us to validate the robustness of our method in the presence of sensor-captured degradations, without relying on synthetic blur assumptions.

Benchmark results are summarized in Table 4. Compared to the baseline methods adapted from [9,10], PIABC achieved the highest PSNR and SSIM values under both 256 × 256 and 128 × 128 patch configurations. Notably, without lens-specific fine-tuning, PIABC attained 30.0258 dB PSNR and 0.8851 SSIM at 256 × 256 resolution, and 30.5716 dB PSNR and 0.8935 SSIM at 128 × 128 resolution. PIABC also achieved LPIPS scores of 0.2143 (256 × 256 patch) and 0.2073 (128 × 128 patch), reflecting reduced perceptual differences relative to the high-resolution ground-truth images. Fine-tuning slightly degraded performance, which is consistent with our observations on DIV2K, suggesting that the pre-learned generalized PSF representations are more robust across diverse real-world variations. This performance drop is likely due to batch-specific adaptation to the limited Canon Test2 subset (50 images). Notably, no distinct visual artifacts were observed, which can be attributed to the patch-based training strategy of the residual U-Net and the pre-filtered PSFs that already encode lens-specific information via Wiener filtering.

Despite known acquisition inconsistencies—having been captured at a different focal length and with a higher magnification—the HR image provided in RealSR-V3 remains a standard reference across recent studies [31,37].

**Figure 8:**
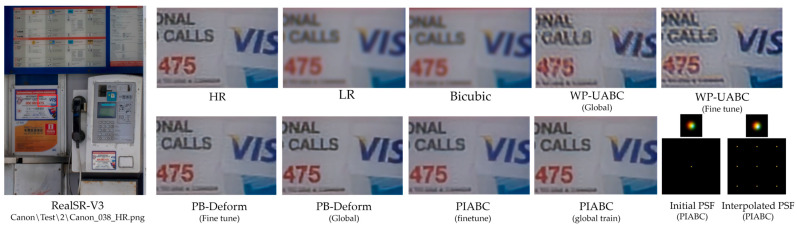
Qualitative comparison on RealSR-V3: Canon2 LR, ID: 038.png; Left: Ground-truth high-resolution image (Canon2-HR); Right: ROI on HR image showing text region (“VIS, CALLS, 475”); Upper: HR, LR, Bicubic, Wiener-prior UABC-net (globally trained and fine-tuned respectively); Lower: Patch-based deformable-net (globally trained and fine-tuned respectively), PIABC (before and after fine-tuning, respectively), PSF (initial CS-sim PSF and interpolated PSF, respectively).

Figure 8 shows the qualitative comparison of restored outputs. PIABC restores sharper edges with fewer artifacts and reduced chromatic shifts. The interpolated PSFs used in our model exhibit smoother spatial structures than those used in other methods, contributing to more effective high-frequency restoration. These results confirm that PSF interpolation, combined with attention-based spatial modeling, enables consistent restoration across both simulated and sensor-acquired real-world images, without requiring prior knowledge of the optical system.

### 4.3. Ablation Study

#### 4.3.1. Analysis of Stage-Wise Effects and Embedding on DIV2K Dataset

To evaluate the effect of positional and PSF embeddings throughout the correction pipeline, we performed stage-wise analysis across four key steps, as summarized in Table 5.

**Figure 9:**
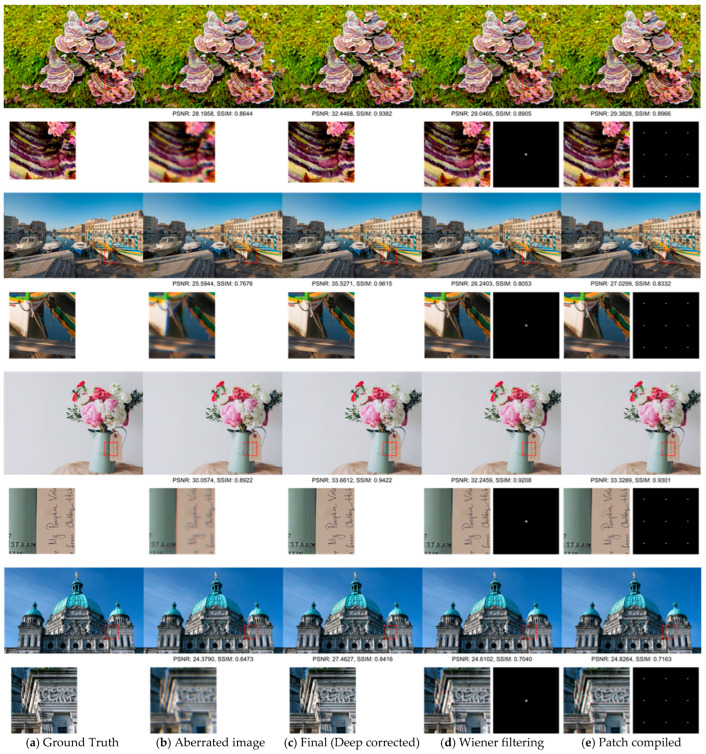
Stage-wise qualitative comparison of restored images: Each row shows a test image from the DIV2K dataset processed through four stages of our restoration pipeline: (**a**) Ground Truth; (**b**) Aberrated image; (**c**) Final output after residual U-Net correction; (**d**) Wiener-filtered image using observed PSFs; (**e**) Patch-wise compilation using interpolated PSFs. Below each row, red-boxed regions are cropped and enlarged for detailed comparison. For (**d**,**e**), the applied PSFs are also visualized on the right. Interpolated PSFs appear smoother and more spatially coherent due to softmax-weighted blending and softplus regularization, which helps reduce aliasing at the cost of some high-frequency sharpness. This facilitates more effective deep correction using residual U-Net.

This experiment was conducted only on the DIV2K dataset, where ground-truth PSF parameters are known. In contrast, the RealSR-V3 dataset does not provide explicit degradation models, making stage-wise attribution infeasible despite the availability of HR-LR pairs. These results quantify the gains at each stage and underscore the contribution of embedding-based PSF interpolation to final restoration quality. Qualitative comparisons corresponding to each stage are illustrated in Figure 9.

Sequential performance gains reveal the relative impact of each module:

- Applying the Wiener filter to the aberrated image improves PSNR and SSIM by 1.4123 dB and 0.0787, respectively.
- Replacing observed PSFs with interpolated PSFs yields a marginal improvement (ΔPSNR = 0.1373 dB, ΔSSIM = 0.0024), suggesting interpolation accuracy aligns closely with actual measurements.
- Compared to using only observed PSFs, the residual U-Net adds a substantial gain of 5.479 dB in PSNR and 0.1161 in SSIM, highlighting the effectiveness of deep refinement.

These results indicate that PSF interpolation meaningfully enhances the performance of the residual U-Net, confirming that the interpolated PSFs contain physically relevant cues that guide the network toward more accurate restorations.

#### 4.3.2. Stage-Wise PSF Modeling and Fine-Tuning Effects on DIV2K Dataset

To examine the effects of PSF embedding and lens-specific fine-tuning on aberration correction performance, we compared four configurations: with/without positional and PSF embedding and before/after fine-tuning. This experiment was performed only on the DIV2K dataset, which provides controlled and consistent PSF labels necessary for isolating embedding and fine-tuning effects. In contrast, RealSR-V3 lacks ground-truth PSFs, making quantitative attribution of such factors infeasible. The results, measured in PSNR and SSIM after both Wiener filtering and residual U-Net deep feature correction, are summarized in Table 6.

**Figure 10:**
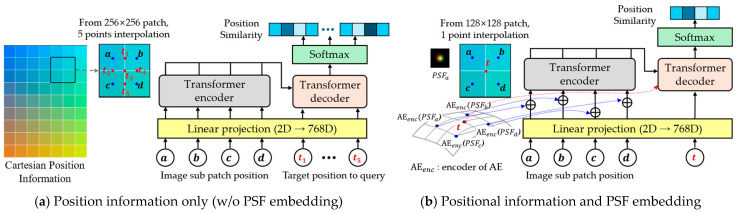
Transformer scheme for image patch input on Cartesian coordinate system: The configurations in (**a**,**b**) correspond to those in Figure 3. The transformer module is illustrated here in a simplified form, as shown in Figure 4; (**a**) None of embeddings are applied, only position information (coordinate) is used with 5 query positions; (**b**) Positional information and PSF embedding used with 1 interpolation query position.; The color shading on the left represents positional information; The letters (a−d, $t_{1}-t_{5}$) follow the patch and query index notation used in Figure 2, Figure 3 and Figure 4; In (**b**), ${AE}_{enc}({PSF}_{j})$ denotes the autoencoder’s encoder module applied to the PSF patch at sub-patch index j (j∈{a,b,c,d}).

Figure 10 illustrates the embedding methods for evaluation.

Across all configurations, PSF embedding consistently led to modest but measurable improvements in PSNR and SSIM. Notably, the best performance was achieved with positional and PSF embedding without fine-tuning. This indicates that embedding high-dimensional PSF features contributes positively to spatial modeling during transformer-based interpolation.

In contrast, the effect of fine-tuning varies depending on the correction stage. When evaluated after using the Wiener filter, fine-tuning slightly improved performance. This suggests that adaptation to lens-specific distortions can benefit early-stage restoration, where correction is shallow and relies on PSF accuracy.

However, after deep correction using the residual U-Net, this trend reversed: models trained without fine-tuning outperformed their fine-tuned counterparts. We interpret this as a possible overfitting effect. Since lens-specific information is already embedded via the PSFs at the Wiener filtering stage, further fine-tuning may introduce redundancy or degrade generalization in the deep learning pipeline.

These findings imply that while embedding enhances restoration through better feature representation, lens-specific fine-tuning should be applied, particularly in architecture where the PSF embedding already encodes optical priors.

#### 4.3.3. Stage-Wise Fine-Tuning Effects on RealSR-V3 Canon 5D3 Dataset

In fine-tuning, additional learning is performed only for data subjected to distortions from the same lens parameters, grouping every 10 images based on presumed similarity.

Unlike conventional SR models, our framework learns specifically for each discrete location, inherently limiting data diversity at each position. Consequently, as shown in Table 7, the combined performance of interpolated PSF and Wiener filtering based on PSF embeddings is reduced after fine-tuning.

PSNR and SSIM show a slight decline compared to the results without fine-tuning, both at the PSF interpolation and residual U-Net stages. This suggests that excessive specialization to limited data induces overfitting, particularly given the small number of available samples (i.e., 50 images in the RealSR-V3 Canon Test2 subset). This trend may also stem from the fact that lens-specific aberration characteristics are already encoded through the PSF-based Wiener filtering, and additional fine-tuning could override the generalizable latent features learned during global training. Consequently, overfitting not only hampers PSF interpolation but also propagates through residual feature refinement stages.

Therefore, while PSF embedding learning, maintaining a wide diversity of PSFs across the latent space appears critical to ensuring generalizable correction.

### 4.4. Results

The performance of PIABC was evaluated on both synthetic (DIV2K) and real-world (RealSR-V3) datasets, allowing for a comprehensive comparison of its effectiveness across controlled and practical scenarios. 

#### 4.4.1. Performance on DIV2K (Synthetic LR)

On the DIV2K dataset, PIABC demonstrated significant improvements over baseline methods. As shown in Table 3, PIABC achieved PSNR values of 31.0044 dB (256 × 256 patch) and 31.4027 dB (128 × 128 patch) before fine-tuning, with SSIM values of 0.9045 and 0.9082, respectively. The corresponding LPIPS values were 0.2036 (256 × 256 patch) and 0.2077 (128 × 128 patch), indicating lower perceptual distortion compared to baseline models.

Compared to the fine-tuned patch-based deformable-net (PSNR 28.2875 dB, SSIM 0.8659, LPIPS 0.2571 for 256 × 256), PIABC yielded up to 9.6% improvement in PSNR, 4.5% in SSIM, and a reduction of 0.0535 in LPIPS.

Figure 7 further illustrates the qualitative superiority of PIABC, showcasing sharper edges, reduced chromatic aberrations, and improved color separation in restored images. These results highlight PIABC’s ability to effectively handle synthetic degradations under controlled conditions, leveraging its interpolative transformer and latent representation space PSF modeling to achieve robust restoration.

#### 4.4.2. Performance on RealSR-V3 (Natural LR)

For the RealSR-V3 dataset, which consists of naturally aberrated low-resolution images captured by a Canon 5D3 DSLR, PIABC maintained strong performance despite the presence of complex sensor noise and optical distortions. Table 4 reports PSNR values of 30.0258 dB (256 × 256 patch) and 30.5716 dB (128 × 128 patch) before fine-tuning, with SSIM values of 0.8851 and 0.8935, respectively. LPIPS values of 0.2143 and 0.2073 (for 256 × 256 and 128 × 128 patches, respectively) further confirm that PIABC restores images with better perceptual fidelity compared to other methods. Compared to the fine-tuned patch-based deformable-net (PSNR 28.9409 dB, SSIM 0.8664 for 256 × 256, and LPIPS 0.2571 to 0.2777 for 256 × 256), PIABC consistently outperformed baselines. Figure 8, PIABC results (lower row, global and fine-tuned) demonstrates PIABC’s ability to restore sharp edges and reduce artifacts in Canon2_LR images, while Figure 8 highlights its effectiveness in recovering fine text details (“VIS CALLS 475”) from a real-world image, achieving significantly sharper text edges and reduced blur artifacts compared to Wiener-prior UABC-net and patch-based deformable-net.

#### 4.4.3. Comparative Analysis and Implications

While PIABC exhibited slightly higher PSNR and SSIM on DIV2K (e.g., PSNR 31.0044 dB vs. 30.0258 dB for 256 × 256 patches) due to the controlled nature of synthetic degradations, its performance on RealSR-V3 remained robust, with only a marginal drop in metrics (e.g., SSIM 0.9045 to 0.8851 for 256 × 256). This consistency underscores PIABC’s generalization ability across diverse degradation types, from synthetic isotropic Gaussian blur to real-world optical aberrations and CMOS sensor noise. The qualitative results further confirm this, as PIABC consistently produced sharper details and fewer artifacts in both datasets (Figure 7 and Figure 8). Notably, the text restoration in Figure 8 emphasizes PIABC’s practical utility in real-world scenarios, where sensor-induced noise and complex aberrations are prevalent, aligning with the needs of CMOS sensor-based systems like the Canon 5D3.

Unlike post-processing approaches that rely on raytraced PSFs from Zemax and lens prescription data [9,10], PIABC takes a pre-processing perspective by simulating chromatic and spatially varying PSFs based on generic optical priors. This is achieved using autoencoder-driven latent embeddings and transformer-based interpolation, without requiring proprietary software or lens-specific metadata. The result is a more adaptable and physically interpretable solution applicable to a wide range of optical systems, including DSLR devices in RealSR-V3.

The effectiveness of this approach is validated by consistent improvements even with low-resolution inputs, as shown in Table 4. Figure 9 and Table 6 further highlight the benefits of our stage-wise embedding and correction process, demonstrating SSIM improvements of up to 4.5% and visibly reduced artifacts in dark regions. Overall, PIABC proves to be a robust computational solution for correcting optical degradation and sensor-related noise, particularly in uncontrolled real-world DSLR imaging environments.

## 5. Discussion

The results demonstrate that point spread function (PSF) interpolation significantly enhances the performance of the residual U-Net (ResU-Net), indicating that interpolated PSFs provide physically relevant cues that guide the network toward improved restoration accuracy. This chromatic-spatial aberration modeling approach, supported by transformer-based techniques, enables robust correction of lens-induced aberrations, particularly effective for lens-induced degradations under spatially smooth degradation conditions; any observed benefit for CMOS sensor data is an indirect result of improved optical correction. Specifically, the transformer interpolation leverages high-dimensional space embeddings derived from an autoencoder (AE), enabling dense and spatially varying PSF modeling. This AE-driven embedding facilitates the transformer’s ability to capture structured optical and sensor behavior, further improving the accuracy of aberration correction. The method’s reliance on PSF-domain interpolation—rather than noisy pixel intensities—enhances optical correction and overall image quality, with any observed reduction of sensor noise being an incidental outcome rather than a directly modeled effect. This was validated on datasets like RealSR-V3, captured with a Canon EOS 5D Mark III (Canon Inc., Tokyo, Japan), (reported as ISO 100 in 90% of images, up to 400 [31]).

The framework’s effectiveness is validated with high-resolution datasets (e.g., DIV2K HR), where fine textures enable precise patch-wise evaluation. Ablation studies on DIV2K and RealSR-V3 confirm the method’s generalization capability, with RealSR-V3’s sensor-acquired low-resolution images (2× zoom) likely exhibiting authentic optical aberrations.

While fine-tuning was expected to improve adaptation to lens-specific distortions, our experiments showed slight performance degradation (Table 3 and Table 4), as pre-filtered PSFs in the Wiener filtering stage already encoded lens-specific information. Consequently, residual U-Net fine-tuning may reinforce batch-specific features at the expense of generalization, especially with limited data diversity. No noticeable fluctuations in validation loss were observed during fine-tuning, and no distinct visual artifacts emerged, which suggests that performance trends might be better interpreted through quantitative metrics rather than visual comparisons. These results underscore the importance of balancing fine-tuning with broad training data to ensure robust generalization.

Although explicit ground-truth PSF maps are unavailable, the imaging setup—combined with prior literature on lens-based distortion in consumer DSLRs—supports the presence of spatially heterogeneous aberrations in Canon2.

This CS-sim PSF strategy captures structured distortion patterns, some of which may be related to sensor characteristics. However, the framework is primarily designed to model and correct lens-induced degradations. Any incidental improvement in sensor-originated artifacts is a secondary effect of improved optical correction.

While the Gaussian-based PSF model enabled efficient interpolation in this study, we recognize that future work should examine how higher-order aberrations might influence restoration accuracy.

Architecturally, CNN–transformer hybrids leverage attention-based feature fusion, while Deformable ResU-Net enhances local spatial adaptability through deformable convolutions. Both aim to improve spatial feature representation, albeit through different mechanisms. In our framework, the transformer interpolator captures spatial and chromatic dependencies during PSF modeling (pre-deblurring), while the residual U-Net refines high-frequency details after physics-based restoration. Adding another transformer in the post-processing stage may duplicate spatial modeling already addressed in the PSF domain, potentially reducing interpretability and increasing redundancy. Our design separates structural alignment and detail enhancement to maintain clarity and robustness.

For post-processing, while recent diffusion-based generative approaches (e.g., DDPM, DDNM) and hybrid CNN–transformer architectures have shown impressive performance in blind restoration, our study adopts a complementary strategy grounded in physically interpretable priors.

## 6. Conclusions

This paper presents a Chromatic-Spatial Simulated PSF (CS-sim PSF) framework for correcting spatially varying aberrations by integrating point spread function (PSF)-domain modeling with dense interpolation techniques. The approach employs autoencoder (AE)-driven latent space embeddings to facilitate transformer-based PSF interpolation, recovering high-resolution outputs with physical plausibility.

Validated across synthetic (DIV2K) and real-world (RealSR-V3) datasets, the method enhances CMOS sensor performance with significant quantitative improvements, without requiring lens-specific calibration. It supports scalable deployment with only low-resolution inputs at inference, affirming the practicality of the proposed technique for computational aberration correction in diverse optical systems.

## 7. Limitations and Future Work

The current framework employs a simplified point spread function (PSF) simulation model that addresses dominant spatial and chromatic degradations but is insufficient to fully capture like coma or astigmatism. Additionally, training relies on high-resolution images, limiting applicability to datasets without high resolution (HR) references. While effective, the use of autoencoder (AE)-driven latent space embeddings for transformer-based PSF interpolation introduces computational complexity due to high-dimensional space processing, potentially restricting real-time sensor applications.

While the Gaussian-based PSF model was chosen for its computational efficiency in this study, its limitations in representing complex wavefront structures are acknowledged. Future work will explore physically grounded models to better capture higher-order aberrations.

We also acknowledge that the current similarity check step does not explicitly account for large field angle PSFs or significant off-axis aberrations, where skew angle variations between adjacent PSFs could reduce the effectiveness of local interpolation. Incorporating more physically informed criteria for similarity checking will be an important area for future study.

Furthermore, the Gaussian-based PSF model, while computationally efficient, may not fully capture complex wavefront structures or higher-order off-axis aberrations.

Future efforts will expand PSF modeling to include a wider range of aberrations and wavefront-based effects. We also plan to optimize the autoencoder–transformer integration to reduce computational cost while preserving the benefits of spatially adaptive PSF interpolation, which is designed to improve optical correction and thereby enhance overall signal quality, potentially reducing the perceptual impact of sensor noise without directly modeling it. Furthermore, developing direct PSF estimation from low-resolution inputs will reduce HR dependency, enhancing applicability under constrained data conditions and improving scalability for practical imaging systems.

## Figures and Tables

**Table 1:** Overview of transformer-based PSF interpolation process.

Stage	Input	Output	Dimension	Description
1	CS-sim PSF ${\tilde{h}}_{ij}^{sp}$	Latent feature ${\overset{˘}{h}}_{ij}^{sp}$	$\left(N,C_{in},\;H_{PSF},\;W_{PSF})\to (N,768\right)$	Patch-level PSFs are flattened and embedded as feature vector.
2	${\overset{˘}{h}}_{ij}^{sp}$, $m_{j}^{sp}$, $c_{i}({=c}_{i}^{p})$	Encoded vector $Q_{i}^{enc}$	$\left(N,768\right),\left(N,2\right)\left(N,2\right)\to (N,768)$	Encoder feature by combining AE output, local PE, and global PE.
3	Query position $t_{il}$	Decoder query vector $q_{il}^{dec}$	(M,2)→(M,768)	Decoder query tokens generated from 2D query positions.
4	$q_{il}^{dec}$, $Q_{i}^{enc}$	Aggregated latent $\mathrm{Feature}\;z_{ij}$	(M,768)→(M,768)	Attention aggregates latent features for each query from encoder outputs.
5	$z_{ij}$	Intermediate PSF ${\hat{h}}_{ij}^{t_{l}}$	$\left(1,\;768)\to (1,\;H_{PSF},\;W_{PSF}\right)$	Decoder reshapes latent feature to generate interpolated PSF patch.
6	${\hat{h}}_{ij}^{t_{l}}$	Final Interpolated PSF ${\hat{H}}_{i}\;\left({\hat{p}}_{i},\;c\right)$	$\left(M,\;H_{PSF},\;W_{PSF})\to (1,\;H_{PSF},\;W_{PSF}\right)$	Weighted sum and smoothing produce final interpolated PSF.

i: sub-patch index; j: query index, j∈{a,b,c,d}; $m_{j}^{sp}$: Sub-patch center position vector (x, y); $c_{i}({=c}_{i}^{p})$: Patch center position vector $\left(x,\;y\right).$ (Note: In embedding-related notations (e.g., embedding indices), regular font is used); p denotes patch; $q_{i,l}^{dec}$, $Q_{i}^{enc}$: Decoder and encoder input features, respectively; $t_{i}$: interpolated PSF target position (final index simplified to i for consistency); $z_{i}$: Aggregated latent feature at target position; ${\hat{H}}_{i}$: Interpolated PSF at target position, obtained via a weighted combination of sub-patch contributions. (j index aggregated via $\alpha_{ij}$, $\gamma_{ij}$ weighted sum.); Self-attention in the encoder and cross-attention in the decoder each use 12 heads.

**Table 2:** Transformer training and architecture configuration.

Component	Specification	Component	Specification	Component	Specification
Embedding Dimension	768	Activation Function	ReLU (PatchEmbed., MLP)	Batch Size	1
Encoder Layers	12	Normalization	LayerNorm (pre-norm config.)	Learning Rates	10^−4^ (model params), 5 × 10^−4^ (spatial weight μ)
Decoder Layers	6	Positional Encoding	Learnable 2D (Cartesian coord.)	Optimizer	Adam, β₁ = 0.9, β₂ = 0.999
Attention Heads	12	Initialization Scheme	Kaiming Uniform (PyTorch default)	Scheduler	StepLR, step size = 1000, decay γ = 0.9
Feed-Forward Hidden Dimension	1536 (MLP ratio = 2.0)	Iterations (global training)	20,000	Loss Function (see Equations (33)–(35))	Total = Rec-PSF + Rec^1^
Dropout Rate	0.1	Iterations (Fine-tuning)	300	Loss Weights	Uniform

Rec^1^: Equation (35) defines the main image reconstruction loss using an $l_{1}$-norm. During implementation, auxiliary terms such as masked region reconstruction and KL (Kullback-Leibler) regularization are added to strengthen spatial learning and stabilize latent representations; Training logs and image outputs are automatically generated during each run to facilitate reproducibility and fine-tuning validation; Data augmentation: Random cropping and horizontal flipping were applied to enhance generalization; Training iterations: 20,000 iterations across 400 training images, using 80 simulated lens models (40 for training and 40 for testing).

**Table 3:** PSNR and SSIM comparison to benchmark works on DiV2K (Average). (a) Five query positions with 256 × 256 patch size; (b) Single central query position with 128 × 128 patch size. (See Figure 3).

Benchmark Model	(a) 256 × 256 Size Patch			(b) 128 × 128 Size Patch		
	PSNR	SSIM	LPIPS	PSNR	SSIM	LPIPS
Wiener-prior UABC-net ^1^ (^4^ global train)	20.9233	0.6273	0.3730	21.3639	0.6418	0.3672
Wiener-prior UABC-net ^1^ (^5^ local finetune)	22.1003	0.6773	0.3427	25.4506	0.7867	0.3382
Patch-based Deformable-net ^2^ (^4^ global train)	26.3358	0.8147	0.2930	25.9587	0.8072	0.3066
Patch-based Deformable-net ^2^ (^5^ local finetune)	28.2875	0.8659	0.2571	27.5892	0.8527	0.2777
PIABC (ours) (before ^6^ fine-tuning) ^3^	**31.0044**	**0.9045**	**0.2036**	**31.4027**	**0.9082**	0.2077
PIABC (ours) (after ^6^ fine-tuning) ^3^	30.7611	0.9000	0.2077	31.2993	0.9079	**0.2058**

^1^ Adapted from Li et al. [9]: Uses a PSF-aware deep network pre-trained on simulated lens data; ^2^ Adapted from Chen et al. [10]: Raytraced PSFs with a deformable CNN for spatial correction; ^3^ Ours (PIABC): Simulated PSF-based correction using transformer-driven latent interpolation; ^4^ globally trained: Trained without considering any variations in dataset (here, DIV2K HR train); ^5^ locally fine-tuned: Perform for every batch of 10 images (5 for training, 5 for testing) assuming those were captured with identical lens (Section 4.1.4); ^6^ Fine-tuning: The same as ^5^ locally fine-tuned; Bold numbers indicate the best performance in each column.

**Table 4:** RealSR-V3 benchmark results on Canon 2 LR (average). (a) Five query positions with 256×256 patch size; (b) Single central query position with 128 × 128 patch size. (See Figure 3).

Benchmark Model	(a) 256 × 256 Size Patch			(b) 128 × 128 Size Patch		
	PSNR	SSIM	LPIPS	PSNR	SSIM	LPIPS
Wiener-prior UABC-net ^1^ (^4^ global train)	25.4307	0.7623	0.3730	24.5512	0.7465	0.3672
Wiener-prior UABC-net ^1^ (^5^ local finetune)	26.4300	0.8109	0.3427	26.3750	0.8157	0.3382
Patch-based Deformable-net ^2^ (^4^ global train)	28.0515	0.8450	0.2930	27.9809	0.8462	0.3066
Patch-based Deformable-net ^2^ (^5^ local finetune)	28.9409	0.8664	0.2571	29.2001	0.8733	0.2777
PIABC (ours) (before ^6^ fine-tuning) ^3^	**30.0258**	**0.8851**	**0.2143**	**30.5716**	**0.8935**	**0.2073**
PIABC (ours) (after ^6^ fine-tuning) ^3^	28.9128	0.8768	0.2208	30.1090	0.8872	0.2120

^1^ Adapted from Li et al. [9]: Uses a PSF-aware deep network pre-trained on simulated lens data; ^2^ Adapted from Chen et al. [10]: Raytraced PSFs with a deformable CNN for spatial correction; ^3^ Ours (PIABC): Simulated PSF-based correction using transformer-driven latent interpolation; ^4^ globally trained: Trained without considering any variations in dataset (here, RealSR Canon 2 LR); ^5^ locally fine-tuned: Perform for every batch of 10 images (5 for training, 5 for testing) assuming those were captured with identical lens (Section 4.1.4); ^6^ Fine-tuning: The same as ^5^ locally fine-tuned; Bold numbers indicate the best performance in each column.

**Table 5:** PSNR and SSIM across different stages on DIV2K (Average): using Cartesian positional information and PSF embedding for 256 × 256 patches.

Process Step	PSNR (dB)	SSIM (dB)
Aberrated image.	23.9758	0.7073
Wiener filter using observed PSFs.	25.3881	0.7860
Compiled image using interpolated PSFs.	25.5254	0.7884
Deeply corrected image.	**31.0044**	**0.9045**

Bold numbers indicate the best performance in each column.

**Table 6:** PSNR and SSIM under four configurations (Average): with/without PSF embedding and before/after fine-tuning. All values are reported after Wiener filtering and residual U-Net correction. Fine-tuning was performed using image batches grouped in units of 10, assuming they share the same lens distortion characteristics.

Fine-Tuning	Embedding Type	^1^ Wiener Restoration		^2^ ResU-net Deep Correction	
		PSNR	SSIM	PSNR	SSIM
Before	^3^ Position only	25.4840	0.7874	30.8906	0.9028
Before	^4^ Positional + PSF embedding	25.5254	0.7884	**31.0044**	**0.9045**
After	^3^ Position only	25.5341	0.7892	30.7736	0.9034
After	^4^ Positional +PSF embedding	**25.5375**	**0.7893**	30.7151	0.9031

^1^ Wiener: restoration using Wiener filtering; ^2^ ResU-Net: Deep correction using the residual U-Net; ^3^ Position only: Uses position information (coordinate) directly, without any explicit positional or PSF embeddings; ^4^ Positional + PSF: Incorporates both coordinate embeddings and PSF feature embeddings. Experiments in this table are conducted only on the DIV2K dataset due to the availability of ground-truth PSFs; Bold numbers indicate the best performance in each column.

**Table 7:** PSNR and SSIM under stage-wise and with/without fine-tuning configuration (average): All values are reported after Wiener filtering and residual U-Net correction. Fine-tuning was performed using image batches grouped in units of 10, assuming they share the same lens distortion characteristics. (a) Five query positions with 256 × 256 patch size; (b) Single central query position with 128 × 128 patch size. (See Figure 3).

Fine-Tuning	Stage	(a) 256 × 256 Size Patch		(b) 128 × 128 Size Patch	
		PSNR	SSIM	PSNR	SSIM
Before	^1^ Wiener	26.6983	0.8038	26.8937	0.8081
Before	^2^ ResU-net	**30.0258**	**0.8851**	**30.5716**	**0.8935**
After	^1^ Wiener	26.6915	0.8038	26.9191	0.8091
After	^2^ ResU-net	28.9128	0.8768	30.1090	0.8872

^1^ Wiener: restoration using Wiener filtering; ^2^ ResU-Net: Deep correction using the residual U-Net; Experiments in this table are conducted on the RealSR(V3)-Canon-LR dataset due to the availability of ground-truth PSFs.; Bold numbers indicate the best performance in each column.

**Figure A1:**
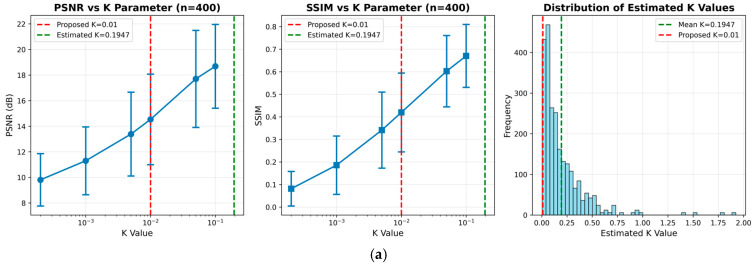

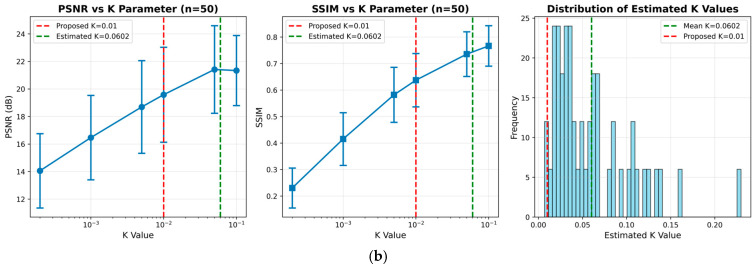
Sensitivity analysis of K value: (**a**) for synthetic dataset DIV2K; (**b**) for real-world datasets RealSR(V3) Canon 2 LR; (Left) PSNR vs. K. (Middle) SSIM vs. K. (Right) Distribution of estimated K values from MMSE criterion.

**Figure A2:**
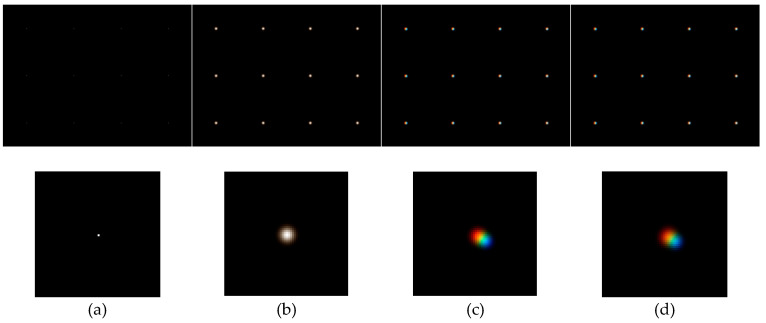
Progressive illustration of a CS-sim-PSF map using 128 × 128-pixel patch. (**a**) Initial isotropic Gaussian PSF; (**b**) addition of Gaussian blur; (**c**) incorporation of chromatic distortion; (**d**) application of elliptical distortion. These steps correspond to the sequential combination described in Equations (4)–(13) in Section 3.2.

## Data Availability

The data presented in this study will be openly available in FigShare at https://doi.org/10.6084/m9.figshare.29328275.

## Appendix A. K Value Sensitivity Analysis for Robust Wiener Filtering

This appendix supplements Section 4.1.6 by providing expanded visualizations and empirical justification for our fixed choice in the Wiener filtering stage. Full-range sensitivity analysis was conducted on both synthetic (DIV2K HR) and real-world (RealSR-V3 Canon2) datasets. Each dataset evaluation used the full test set to ensure statistical reliability: 400 images for DIV2K HR and 50 images for RealSR-V3.

To rigorously validate our empirical choice of K = 0.01, we conducted extensive sensitivity analysis across two distinct datasets representing different degradation characteristics. K values were evaluated, and performance was measured using PSNR and SSIM, with error bars representing 95% confidence intervals across full test sets (*n* = 400 and *n* = 50, respectively).

**Table A1:** Quantitative results for K sensitivity analysis based on full test sets.

Dataset	K Value	PSNR (dB)	SSIM
DIV2K HR Train	0.0002	9.81 ± 2.04	0.0815 ± 0.0765
	0.0010	11.30 ± 2.65	0.1857 ± 0.1294
	0.0050	13.39 ± 3.28	0.3413 ± 0.1684
	0.0100	14.53 ± 3.54	0.4197 ± 0.1747
	0.0500	17.70 ± 3.79	0.6026 ± 0.1580
	0.1000	18.69 ± 3.20	0.6703 ± 0.1397
RealSR (V3) Canon 2 LR	0.0002	14.06 ± 2.70	0.2305 ± 0.0754
	0.0010	16.47 ± 3.06	0.4152 ± 0.0994
	0.0050	18.69 ± 3.37	0.5819 ± 0.1038
	0.0100	19.58 ± 3.45	0.6372 ± 0.1006
	0.0500	21.42 ± 3.18	0.7355 ± 0.0843
	0.1000	21.34 ± 2.54	0.7664 ± 0.0763

For each K value, we measured PSNR and SSIM metrics across multiple test samples, with error bars representing 95% confidence intervals. While the optimal K varies with data characteristics (K = 0.1947 for DIV2K and K = 0.0602 for RealSR), K = 0.01 provides stable and competitive results (within 2–4 dB of the optimal) across both domains.

**Table A2:** Comparison of optimal and empirical K values across datasets. The deviation factor quantifies the ratio between the MMSE-optimal and fixed.

Dataset	Optimal K	K = 0.01 Performance	Deviation Factor
DIV2K HR Train	0.1947	PSNR: 14.53 ± 3.54 dB, SSIM: 0.4197 ± 0.1747	19.5×
RealSR (V3) Canon 2 LR	0.0602	PSNR: 19.58 ± 3.45 dB, SSIM: 0.6372 ± 0.1006	6.0×

Note: The estimated K values are obtained using an MMSE-based method that leverages Laplacian variance to estimate noise power (Immerkaer, 1996 [38]), widely adopted for unsupervised Wiener filter tuning.

The results demonstrate the following insights:

1. Validity of K = 0.01:
  - The histogram of MMSE-estimated K values shown in Figure A1 (a, right) and Figure A1 (b, right) shows a dominant concentration in the range of 0.02 to 0.12, with a mean of 0.1947 and 0.0602, respectively. This supports the view that our empirical choice (K = 0.01) is a conservative yet well-grounded setting.
  - Our empirical choice of K = 0.01 lies near the lower bound of this range, representing a conservative yet reasonable selection grounded in signal preservation.
2. Sensitivity Trends:
  - As K increases from 0.0002 to 0.1000, both PSNR and SSIM improve steadily:
    – DIV2K: PSNR improves from 9.81 dB to 18.69 dB, SSIM from 0.0815 to 0.6703,
    – RealSR: PSNR improves from 14.06 dB to 21.34 dB, SSIM from 0.2305 to 0.7664.
  - It shows a robust, quasi-logarithmic relationship between K and restoration quality.
3. Robustness of Fixed K:
  - Despite not being the MMSE-optimal, K = 0.01 yields strong intermediate performance across datasets.
  - RealSR shows only 1.76 dB loss (vs. optimal K); DIV2K shows 4.16 dB loss.
  - The consistent error bar trends validate the reliability of this fixed setting across real and synthetic domains.

Therefore, the empirical selection of K = 0.01 represents a robust engineering compromise that maintains strong restoration performance across both synthetic and real-world scenarios. Its stability, combined with the deep network’s ability to compensate for residual noise, justifies its use as a fixed hyperparameter.

## Appendix B. Illustration of CS-sim-PSF

To complement Section 3.2, we provide an illustration of the CS-sim-PSF map generation process using a 128 × 128-pixel patch. Figure A2 shows four cropped PSF patches from the upper-right corner of the image, each depicting a different stage of simulated optical degradation: (a) isotropic Gaussian blur, (b) additional blur, (c) chromatic distortion, and (d) elliptical distortion. The lower image shows a zoomed-in view of the PSFs to illustrate the progression of degradation steps more clearly.

These progressive degradations are generated using the parametric models defined in Equations (4)–(13) of Section 3.2. In particular, the PSF at each pixel is defined as a composition of isotropic and elliptical Gaussian functions, along with chromatic displacements, based on this formulation.

This formulation allows us to approximate local aberration patterns, including both chromatic displacement and elliptical distortions, without requiring direct wavefront measurements or global Zernike decompositions. It effectively captures spatially varying degradations and supports patch-wise training of the restoration model. While our method does not explicitly validate PSFs against Zernike polynomials, future work may explore such integration to bridge traditional optical models and data-driven frameworks.
